# Better Executive Functions Are Associated With More Efficient Cognitive Pain Modulation in Older Adults: An fMRI Study

**DOI:** 10.3389/fnagi.2022.828742

**Published:** 2022-07-07

**Authors:** Katharina M. Rischer, Fernand Anton, Ana M. González-Roldán, Pedro Montoya, Marian van der Meulen

**Affiliations:** ^1^Department of Behavioural and Cognitive Sciences, Institute for Health and Behaviour, University of Luxembourg, Esch-sur-Alzette, Luxembourg; ^2^Cognitive and Affective Neuroscience and Clinical Psychology, Research Institute of Health Sciences, Balearic Islands Health Research Institute, University of the Balearic Islands, Palma, Spain

**Keywords:** pain modulation, distraction, aging, executive functions, prefrontal cortex, fMRI, gray matter volume

## Abstract

Growing evidence suggests that aging is associated with less efficient endogenous pain modulation as demonstrated by reduced conditioned pain modulation, and that these changes may be mediated by differences in frontal functioning. Yet, little is known about potential age-related changes in cognitive pain modulation, such as distraction from pain. In a first session, 30 healthy young (19–35 years) and 30 healthy older (59–82 years) adults completed a battery of neuropsychological tests. In a second session, we acquired functional brain images while participants completed a working memory task with two levels of cognitive load (high vs. low) and concurrently received individually adjusted heat stimuli (warm vs. painful). In both age groups, completing the high load task was associated with a significant reduction in the perceived intensity and unpleasantness of painful stimuli and a reduction in activation of brain regions involved in pain processing. Group comparisons revealed that young adults showed a stronger de-activation of brain regions involved in pain processing during the high load vs. the low load task, such as the right insula, right mid cingulate cortex and left supramarginal gyrus, compared to older adults. Older adults, on the other hand, showed an increased activation in the anterior cingulate cortex during the high load vs. low load task, when compared to young adults. Covariate analyses indicated that executive functions significantly predicted neural pain modulation in older adults: Better executive functions were associated with a more pronounced de-activation of the insula, thalamus and primary somatosensory cortex and increased activation of prefrontal regions during the high vs. low load task. These findings suggest that cognitive pain modulation is altered in older age and that the preservation of executive functions may have beneficial effects on the efficacy of distraction from pain.

## Introduction

A steadily increasing life expectancy has led to a growing clinical and empirical interest in age-related changes in the peripheral and central processing of pain and their subsequent effects on pain perception and pain modulation (Farrell, 2012). In an attempt to uncover potential mechanisms behind older adults’ increased risk of developing chronic pain (Prostran et al., 2016; Domenichiello and Ramsden, 2019), researchers have increasingly focused on age-related changes in the brain that may affect pain processing and endogenous pain modulation. One such region of interest is the prefrontal cortex (PFC), a structure that is heavily involved in the top-down modulation of pain (Ploghaus et al., 2003; Wiech et al., 2006, 2008), and that is particularly affected by age-related atrophy in gray matter (GM) volume (Crivello et al., 2014). A growing number of studies suggest that the efficacy of endogenous pain modulation is mediated by interindividual differences in frontal functioning as reflected by differences in executive functions (EFs) or regional GM volume in the PFC (Benedetti et al., 2004; Marouf et al., 2014; Coppieters et al., 2015; Ickmans et al., 2015; Meeus et al., 2015; Zhou et al., 2015a; Lithfous et al., 2019; Palermo et al., 2019; Bunk et al., 2020, 2021; Rischer et al., 2020), and point to a particularly strong link between cognitive inhibition abilities and endogenous pain modulation (Marouf et al., 2014; Ickmans et al., 2015; Palermo et al., 2019; Rischer et al., 2020).

However, the majority of these studies either tested the effects in patients with dementia or Alzheimer’s disease (who show substantially larger impairments in frontal functioning than healthy older adults; Benedetti et al., 2004; Palermo et al., 2019; Bunk et al., 2021), in chronic pain patients (Coppieters et al., 2015; Ickmans et al., 2015; Meeus et al., 2015) (who may show altered pain processing; May, 2008) or did not specifically focus on the aging brain (Rischer et al., 2020). Moreover, most of these studies assessed endogenous pain modulation using conditioned pain modulation (CPM) or temporal summation (TS) paradigms (Marouf et al., 2014; Coppieters et al., 2015; Ickmans et al., 2015; Meeus et al., 2015; Lithfous et al., 2019; Bunk et al., 2020, 2021), leaving open the question of how other forms of endogenous pain modulation, such as cognitive distraction from pain (Moont et al., 2010), are affected by aging. Distraction is a common and intuitive pain coping strategy (Goubert et al., 2004) that is preferably used among older adults suffering from chronic pain (Lansbury, 2000), increasingly employed in clinical settings to complement pain medication (Malloy and Milling, 2010; Li et al., 2011), and potentially works through a different physiological mechanism than CPM or TS (Moont et al., 2010).

To our knowledge, only two studies to date have directly investigated age-related changes in distraction from pain in healthy older adults (Zhou et al., 2015a; González-Roldán et al., 2020a). While the results of both studies point to age-related differences in the electrophysiological correlates of distraction from pain, evidence for changes in the behavioral distraction effect size is less clear, probably due to differences in task demand and pain intensity. In the present study, we aimed to investigate potential age-related changes in the behavioral and neural correlates of cognitive distraction from pain and their relationship to prefrontal functioning as indexed by executive functions and gray matter volume.

We expected that distraction from pain should lead to a de-activation of brain regions involved in pain processing such as the insula, thalamus, postcentral gyrus (primary somatosensory cortex) and areas in the cingulate cortex (see e.g., Frankenstein et al., 2001; Bantick et al., 2002; Coen et al., 2008). We furthermore expected an increase in activation in frontal areas and areas in the cingulate cortex during distraction from pain, as these brain areas have been implicated in mediating the neural distraction effect in previous studies (Petrovic et al., 2000; Frankenstein et al., 2001; Bantick et al., 2002; Valet et al., 2004).

We expected to find that individuals with worse EFs, specifically worse cognitive inhibition abilities, and more GM atrophy in the PFC (as well as in other areas associated with pain modulation) would show a smaller neural distraction effect, i.e., a weaker reduction in activation of pain-related brain areas, than individuals with better EFs, and that this relationship should be especially pronounced for older adults. Finally, we expected worse EFs to be associated with a smaller increase in activation in frontal regions during distraction, in older adults.

## Materials and Methods

### Participants

Young (YA) and older adults (OA) were recruited through advertisement at the University of Luxembourg (targeted at regular students and senior guest students), interviews in local media and advertisements at organizations and services for senior citizens in Luxembourg between July 2018 and September 2019. All participants were in good health and had normal or corrected-to-normal vision. General exclusion criteria were the presence of major depression, and any psychiatric or neurological disorders, including neuropathy. Other exclusion criteria included substance or alcohol abuse, and injuries or large tattoos on the volar surface of their left arm where heat stimuli were applied. Participants self-reported to be free from acute or chronic pain. Participants with mild intermittent pain (e.g., occasional back or neck pain) could take part in the study as long as they were pain free on the day of the test session and did not take pain medication on a regular basis. Furthermore, none of the participants presented contraindications for an MRI scan (i.e., epilepsy, claustrophobia, pregnancy, metal implants, heart pacemakers or insulin pumps). Participants were requested not to take any pain medication or other drugs known to have an impact on sensory perception or cognition prior to the experimental sessions. Given that the University of Luxembourg is a trilingual university accommodating English-, German-, and French-speaking students, all questionnaires, neuropsychological tests, and experimental instructions were available in English (28.3%), German (66.7%), and French (5.0%).

A total of 33 younger and 35 older participants were recruited for the study. One YA had to discontinue the fMRI session due to skin irritation at the site of pain stimulation; a further two YA were excluded from the analyses due to technical problems with the thermal stimulator during data acquisition. Two OA completed a neuropsychological assessment but were not re-invited to the fMRI session due to the daily intake of pain medication or the excessive daily consumption of alcohol. Two OA were excluded from the analyses because of abnormal brain lesions (as established by a neuroradiologist), and one OA had to discontinue the fMRI session due to difficulties to follow task instructions. Thus, a final sample of 30 young (11 male; age: *M* = 26.7, *SD* = 4.20; age range: 19–35 years) and 30 older (16 male; age: *M* = 67.73, *SD* = 6.50; age range: 59–82 years) participants were included in the analyses.

Participants received a compensation of €40 (in form of gift vouchers) for their time and effort and had the chance to win a gift voucher worth €80 in a prize draw. In addition, they could request a CD with images of their brain and a report of their performance in the neuropsychological assessment. The study was conducted in accordance with the Declaration of Helsinki and ethical approval was obtained from the Ethics Review Panel of the University of Luxembourg and the Luxembourg national ethics committee for research (CNER). All participants gave their informed consent at the start of the study.

### Procedure

Participants were invited to two experimental sessions: a neuropsychological session (s_1_) at the University of Luxembourg, and following this, an fMRI session (s_2_) at a hospital (Hôpitaux Robert Schuman) in Luxembourg City (s_2_-s_1_: *M (days)* = 23.15, *SD* = 44.13). During the first session, participants completed a neuropsychological test battery targeting neurocognitive functions and their thermal pain thresholds were measured (data not reported here). In the fMRI session, we assessed distraction from pain with a validated paradigm (Buhle and Wager, 2010; González-Roldán et al., 2020a; Rischer et al., 2020; Tabry et al., 2020) while acquiring functional magnetic resonance images. In this paradigm, innocuous warm and painful heat stimuli were administered to the participants’ left arm while they performed a low or high load working memory task. Importantly, given that age has been associated with altered pain sensitivity (Lautenbacher et al., 2017) and working memory is known to show age-related decline (Bopp and Verhaeghen, 2020), we calibrated the intensity of the thermal stimuli and task speed for each participant. At the start of each session, participants completed a brief questionnaire about their drug consumption (nicotine, caffeine, medication) in the days before the test session, as well as the Positive and Negative Affect Schedule (PANAS) (Watson et al., 1988) to assess their current positive affect and negative affect (scale range for positive and negative affect subscales: 10–50; a higher score indicates more positive or negative affect). At the end of the fMRI session, participants completed a post-experimental questionnaire about the perceived task difficulty, their ambition to perform well, task-induced stress and the perceived distractive effects of the tasks (see Supplementary Materials for more details). The neuropsychological session lasted on average between 1.5 and 2 h, and the fMRI session lasted 2 h.

### Neuropsychological Session

#### Questionnaires

Participants completed a demographic questionnaire about their health status (including questions about ongoing medical/psychological treatments, chronic pain and intake of pain medication) as well as the Depression Anxiety and Stress Scale (DASS-42; scale range: 0–126; a higher score indicates more emotional distress) (Lovibond and Lovibond, 1995) to establish that they met inclusion criteria (e.g., absence of major depression or chronic pain). In addition, participants provided information on their education level and completed the Edinburgh Handedness Inventory (EHI; scale range: –100 to 100; a higher score indicates the more frequent use of the right hand) (Oldfield, 1971).

To assess pain-related cognitions, they also filled out the Fear of Pain Questionnaire (FPQ-III; scale range: 30–150; a higher score indicates more fear of pain) (McNeil and Rainwater, 1998), the Pain Catastrophizing Scale (PCS; scale range: 0–52; a higher score indicates more pain catastrophizing) (Sullivan et al., 1995) and the Pain Vigilance and Awareness Questionnaire (PVAQ; scale range: 0–80; a higher score indicates more vigilance to pain) (McCracken, 1997). Validated versions for all questionnaires were available in all three languages; the French version of the FPQ-III was partly adapted from a validated short version (Albaret et al., 2004) and the remaining items were translated by a French native speaker. The French version of the PVAQ (Desrochers et al., 2009) was based on a 5-point scale and scores were later transformed to a 6-point scale, to be compatible with the other language versions.

#### Neuropsychological Tests

Participants completed a battery of neuropsychological tests that were administered in a pseudorandomized order. Relevant tests included the Mini Mental State Examination (MMSE) (Folstein et al., 1975) to screen for cognitive impairments, the Stroop Color-Word Test-Victoria version (Spreen et al., 1998) to quantify response inhibition abilities, the digit span test (from the WAIS-IV) (Wechsler, 2008) as an index of working memory, and a computerized version of the flanker task, adapted from Wylie et al. (2009) to measure interference control and selective attention. In addition, they completed the Trail Making Test (TMT) to assess processing speed (TMT-A) and higher level cognitive skills, such as task-set inhibition and cognitive flexibility (TMT- B) (Bowie and Harvey, 2006). The difference in time taken to complete the TMT-B relative to the TMT-A was used as a measure for central executive functioning (McMorris, 2015). A smaller flanker and Stroop effect and TMT difference score indicate better performance (i.e., executive functions) whereas a higher digit span score (range: 0–48) indicates better working memory abilities.

### fMRI Session

Upon arrival, the experimenter briefly explained the study set-up without revealing the actual aim of the study (i.e., to investigate age-related differences in distraction from pain). They were informed that better task performance in the experiment would increase their chances of winning the €80 gift voucher (by including a higher number of ballots in the prize draw) as a monetary incentive has been shown to increase task engagement (Verhoeven et al., 2010); in reality, all participants had an equal chance to win the gift voucher. Participants then practiced a low (control condition) and high load (distractor condition) version of a working memory task on a desktop computer in the MRI control room (a description of the tasks can be found below). Following this, participants completed an on-line calibration of the presentation speed for the high load task to account for age-related differences in perceived task difficulty (Rischer et al., 2020; see Supplementary Materials for a detailed description of the algorithm). After this, participants underwent a calibration procedure (further described below) to select one individually adjusted non-painful warm and one moderately painful temperature for the distraction paradigm. Participants were then installed in the scanner to complete the pain distraction paradigm. Visual stimuli were presented on an MRI compatible LED monitor (Optostim, Cologne, Germany) placed directly behind the MRI scanner bore. Participants could see the screen via an angled mirror mounted on the head coil. If necessary, a version of the experimental task with a larger font size was used. The thermal stimulation was triggered, and the working memory task was presented using E-Prime 2 (Psychology Software Tools Inc, Pittsburgh, PA, United States).

#### Thermal Stimulus Calibration

In the calibration procedure, participants were asked to rate the perceived intensity of a series of thermal stimuli on a 200-point visual analog scale (VAS), with the pain threshold in the middle (corresponding to a VAS rating of 100). The ratings were subsequently interpolated and temperatures corresponding to an intensity of 60 points (innocuous warm stimuli) and 140 points (moderately painful stimuli) were determined (more details of the procedure can be found in the Supplementary Materials).

#### Distraction Paradigm

**FIGURE 1:**
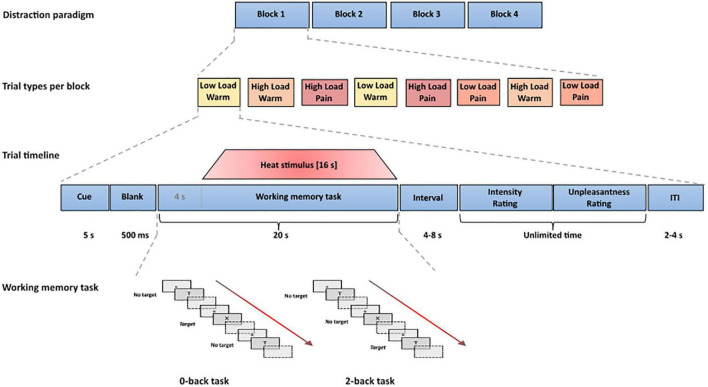
Experimental design and trial timeline. Each trial consisted of a cue word signaling the upcoming task, followed by the task (20 s) with thermal stimulation starting 4 s after task onset and lasting for 16 s. After each task, participants were asked to rate the intensity and unpleasantness of the thermal stimulation. The low and high load task consisted of a 0-back and 2-back working memory task, respectively. Each letter was presented for 500 ms, preceded by a fixation cross (250 ms) and followed by a blank inter-character interval. The duration of the inter-character interval was individually adjusted in a calibration phase prior to, and throughout, the experiment to account for differences in task difficulty by adjusting the task speed.

Participants completed 16 low load and 16 high load trials of a working memory task while receiving thermal stimuli on their left forearm (see Figure 1). Following each trial, participants rated the intensity and unpleasantness of the stimuli on 200-point VASs. In half of the trials (eight low load and eight high load task trials) a non-painful warm stimulus was presented, in the other half a painful heat stimulus. There were thus four different types of trials: warm/low load, warm/high load, pain/low load and pain/high load. The total of 32 trials were divided into four blocks (eight trials per block; two trials per trial type). The completion of a block took about 8 min. In between blocks, acquisition of fMRI images was stopped, the experimenter briefly checked on the participant via an intercom, and the participant could take a short break if necessary. The different types of trials within a block were presented in randomized order, with the constraint that no more than two high load or two painful trials were presented in a row.

#### Trial Timeline

Each trial started with the presentation of a cue word (presented for 5,000 ms) that signaled the upcoming n-back task (either “X-target” for the low load task or “2-back” for the high load task), followed by a blank screen (500 ms) and then by the n-back task (20 s, with the thermal stimulation commencing 4 s after task onset and ending simultaneously with the task), an interval of 4–8 s (average: 6 s) and two rating scales (unlimited time; with an interval of 500 ms between the intensity and unpleasantness scale). After an intertrial interval of 2–4 s (average: 3 s) displaying a fixation cross, the next trial started (see Figure 1).

#### Working Memory Task

A letter n-back task was used as the working memory task. In each trial, participants were presented with a series of letters. In the high load (2-back) condition, participants had to indicate for each letter whether it was the same as the letter presented two steps back in the sequence or not. The low load (0-back) task required participants to indicate whether the current letter was an “X” or not, and thus only differed from the high load condition in terms of the instructions (see Figure 1). A more detailed description of the task settings can be found in the Supplementary Materials.

#### Thermal Stimulation

Thermal stimuli of 16 s duration were administered to the lower left forearm, with an MRI compatible 3 × 3 cm Peltier thermal stimulator (Somedic AB, Sösdala, Sweden). Stimuli consisted of a plateau phase of 10 s and ramp-up/ramp-down phases of 3 s each. For painful stimuli, the slope of the ramp-up/down phases was set to 5°C/s and for non-painful stimuli to 3°C/s. Baseline temperature was set to 34°C.

#### Visual Analog Scale Ratings

Participants rated the intensity and unpleasantness of the thermal stimuli on 200-point VASs using a hand-held button box (Current Designs, Philadelphia, PA, United States) during both a nociceptive calibration procedure and the main experimental task. The scales ranged from 0 (“no warmth”) via 100 (“just pain”) to 200 (“unbearable pain”), and the unpleasantness scale from 0 (“very pleasant”) via 100 (“neutral”) to 200 (“very unpleasant”).

#### fMRI Acquisition and Preprocessing

Whole-brain functional images were acquired on a 1.5T MRI system (Magnetom Aera, Siemens, Germany) with a 20-channel head coil. Before participants were placed in the scanner, they were provided with in-ear hearing protection and headphones to reduce scanner noise and their head position was stabilized with foam cushions to restrict movements. An intercom was used to communicate with participants during the breaks. The scanning protocol consisted of the acquisition of a magnetic fieldmap, four functional runs of the distraction paradigm (corresponding to the 4 blocks with eight trials each), a 6 min resting-state functional run, several anatomical scans and a diffusion weighted (DTI) scan.

All functional images were acquired using a susceptibility-weighted EPI sequence (TR/TE = 2490/31 ms; flip angle = 80°; FOV = 192 mm; matrix size = 64 × 64 pixels). Thirty-three transversal slices were acquired in interleaved (descending) order, with a 4 mm thickness and a 10% gap, yielding a voxel size of 3 × 3 × 4 mm. High-resolution anatomical images were acquired using a T1-weighted MP-RAGE sequence (176 sagittal slices, TR/TI/TE = 1,900/913/2.33 ms; flip angle = 9°; voxel dimensions = 0.9 mm isotropic; FOV = 230 mm); a FLAIR sequence (160 sagittal slices; TR/TI/TE = 4500/1800/284 ms; flip angle = 120°; voxel dimensions = 0.9 mm isotropic; FOV = 234 mm; slice thickness = 0.95 mm); and a T2-weighted turbo spin echo (TSE) pulse sequence (28 transversal slices; TR/TE = 6000/94 ms; flip angle = 150°; voxel dimensions = 0.6 × 0.6 × 4 mm; FOV = 230 mm; slice thickness = 4 mm). These structural images were assessed for age-related atrophy, vascular alterations, and other brain lesions by a neuroradiologist.

Functional images were pre-processed and analyzed using SPM12 (Wellcome Centre for Human Neuroimaging, London, United Kingdom). First, bad slices were identified and repaired with the ArtRepair toolbox (Mazaika et al., 2009) and subsequently slice-time corrected (all slices resampled to the acquisition time of the first slice). We then applied motion and distortion correction (realignment and unwarping using the EPI-based fieldmap image) and co-registered the T1-weighted anatomical scan to the mean resliced and segmented functional image. These were then smoothed with an isotropic Gaussian kernel of 4 mm full width at half maximum, after which additional correction for remaining motion artifacts in preprocessed volumes using the Motion Adjustment and Despike options in the ArtRepair toolbox (Mazaika et al., 2009) was applied. Finally, we normalized the structural image to the MNI template, applied the normalization parameters to all functional images and smoothed the images again (FWHM = 7 mm; note that smoothing of FWHM = 4 and 7 mm is equivalent to a FWHM = 8 mm).

Gray matter volume was extracted using the Computational Anatomy Toolbox (CAT 12.7) (Gaser and Dahnke, 2016). T1-weighted images were corrected for magnetic field inhomogeneities, spatially normalized using the shooting algorithm, and segmented into GM, white matter (WM), and cerebrospinal fluid (CSF), using the default parameter settings in the CAT 12.7 toolbox. The resulting GM segments were then smoothed (FWHM = 8 mm).

### Statistical Analyses

#### Behavioral Distraction Effect

Statistical analyses were performed using SPSS 25 (IBM SPSS Statistics). Age-related differences in demographic and neuropsychological characteristics were evaluated using two-tailed independent samples t-tests. The magnitude of the distraction effect (i.e., a reduction in VAS pain ratings) was assessed with a repeated measures ANOVA with the within-subject factors *temperature level* (warm vs. pain) and *task difficulty* (low load vs. high load) and the between-subject factor *age group* (YA vs. OA). We furthermore obtained a behavioral distraction effect score for intensity (DE-I) and unpleasantness ratings (DE-U) by computing the difference in VAS scores for painful compared to warm stimuli in the low load compared to the high load condition, i.e., DE-I (or DE-U) = (low load/pain – low load/warm) – (high load/pain – high load/warm) in correspondence with the first-level fMRI contrast that we used to assess the neural distraction effect. We used these difference scores to assess potential relationships between the behavioral distraction effect and pain-related cognitions and executive functions using Pearson correlations with bias-corrected and accelerated bootstrapping (1,000 samples). Note that we did not assess the effects of pain on task performance (pain-induced task interference) as we (partially) controlled for differences in task performance by continuously adjusting task speed. We used a significance level of α = 0.05 for all analyses. In case of multiple comparisons, we used a Bonferroni-corrected α. Partial eta squared ($\eta_{\text{p}}^{2}$) effect size measures are reported for significant effects in the ANOVA models, where 0.01 represents a small effect, 0.06 represents a medium effect and 0.14 represents a large effect (Cohen, 1973).

#### Neural Pain Response, Distraction Effect and Mechanism

The first level design matrix of each participant included four regressors of interest (corresponding to the four trial types: warm/low load, warm/high load, pain/low load, pain/high load; comprising the 16 s thermal stimulation interval), as well as a regressor for the rating duration and a session constant as regressors of no interest. Regressors of interest in the first level design matrices were convolved with the canonical Hemodynamic Response Function (HRF), and contrast images related to the four trial types were computed for each participant. To analyze age-related differences in brain activation on the group level, we entered the four contrast images of each participant into a flexible factorial model with the factors *age group*, *temperature level*, and *task difficulty.*

To verify that the painful stimuli indeed elicited pain-related activation in the brain, we first created a *pain* > *warm* contrast, collapsing the two task conditions and two groups. To examine the *neural distraction effect*, i.e., a de-activation in pain-related neural activity in the high load compared to the low load condition, we computed the following contrast: (*pain* > *warm) _low load_* > (*pain* > *warm)_high load_*. We also examined the *neural distraction mechanism*, targeting any areas that may be involved in driving the distraction effect and thus were more active during the high vs. the low load condition, with the following contrast: (*pain* > *warm)_high load_* > (*pain* > *warm)_low load_*. In addition, we compared differences in neural activation between both age groups by computing the contrast YA > OA and OA > YA for the neural pain response and the neural distraction effect and mechanism. These contrasts were masked with the contrast map obtained for the neural pain response, distraction effect or distraction mechanism for YA (when contrasting YA > OA) or OA (when contrasting OA > YA) at *p* < 0.005. For all contrasts, we first conducted an exploratory search using an uncorrected threshold of *p* < 0.005 with at least 10 contiguous voxels. We then applied a cluster-level FDR correction at *p* < 0.05. All activation tables report both exploratory and corrected results. Anatomical labels of activated clusters were determined automatically using the Anatomy Toolbox version 2.2b (Eickhoff et al., 2005).

#### Neural Effects and Executive Functions

To assess the relationship between executive functions and the neural distraction effect and mechanism, we added the flanker effect score, Stroop effect score, TMT difference score and total digit span score as covariates to separate two-samples *t*-test models. In addition, we exploratorily assessed whether there was any relationship between the neural distraction effect and the behavioral distraction effect measures for both age groups. We used an uncorrected cluster-defining threshold of *p* < 0.001 with at least 20 contiguous voxels, and effects were considered significant if they survived a cluster-level FDR correction at *p* < 0.05. To visualize the correlation between executive functions and the neural effects with scatterplots, we extracted the parameter estimates (summary time course) of the clusters of interest using the MarsBaR toolbox (Brett et al., 2002).

#### Gray Matter Volume and Distraction Effect

Differences in GM volume between age groups were analyzed with the Computational Anatomy Toolbox (CAT 12.7) (Gaser and Dahnke, 2016). The pre-processed and smoothed GM segments were entered in a second level two-samples *t*-test model in SPM 12, with the extracted total intracranial volume (TIV) for each participant as nuisance factor and with an absolute masking threshold of 0.2 to avoid possible edge effects between GM and WM or CSF (Farokhian et al., 2017). Group differences were assessed with a whole-brain FWE corrected threshold of *p* < 0.05. To examine whether the behavioral distraction effect size was associated with differences in GM volume, we entered distraction effect scores for the intensity and unpleasantness scale (DE-I and DE-U) as covariates to two separate t-test models (with a cluster-defining threshold of *p* < 0.001 with *k* ≥ 20, and cluster-level FDR correction at *p* < 0.05).

In addition, we examined the role of total GM volume (controlled for TIV) as a potential mediator of age-related differences in the behavioral distraction effect size, and neural distraction effect and mechanism, by adding normalized GM volume scores as covariates to the repeated measures ANOVA (to assess the influence of total GM volume on the behavioral distraction effect size) and as nuisance factor to the *t*-test model of the neural distraction effect and mechanism (to assess the influence of total GM volume on age-related differences in neural activations).

## Results

### Group Characteristics

Independent sample *t*-tests revealed that YA and OA did not differ significantly regarding their MMSE scores, indicating that our older sample did not show any general cognitive impairment. However, older adults showed a significant age-related decline in most executive functioning tests (see Table 1). Groups did not differ with respect to emotional distress symptoms (as measured with the DASS-42), but older adults reported significantly less negative pain-related cognitions (FPQ-III, PCS) and more positive affect (PANAS) than young participants in both sessions. Distraction paradigm settings, information about medication intake and the evaluation of the paradigm by the participants in the post-experimental questionnaire can be found in the Supplementary Tables 1–3.

**TABLE 1:** Group characteristics.

	YA		OA			
	*Mean*	*SD*	*Mean*	*SD*	*t-statistic*	*P-value*
***Demographics***						
Education (years)^a^	16.30	2.18	14.22	3.74	–8.38	<0.001
EHI	73.17	42.46	91.37	18.92	–2.15	0.038
MMSE	29.40	0.77	29.13	1.01	1.15	0.254
DASS-42 total	16.37	13.13	10.93	11.59	1.70	0.095
***Pain-related cognitions***						
FPQ-III total^b^	81.00	17.06	60.17	17.58	4.62	<0.001
PCS total	17.83	9.02	10.77	10.22	2.84	0.006
PVAQ total^c^	35.14	14.75	33.87	14.53	0.34	0.737
***Executive functions***						
TMT difference score^d^	26.32	14.97	40.03	20.01	–3.00	0.004
Stroop effect^d^	7.26	5.16	13.46	4.19	–5.06	<0.001
Digit span (total)	29.43	6.17	26.00	4.55	2.45	0.017
Flanker effect^d^	41.46	40.49	67.31	60.02	–1.93	0.059
***Affective state (PANAS)***						
Session 1: Positive	30.57	4.07	36.13	4.07	–4.42	<0.001
Session 2: Positive	31.23	4.85	35.03	6.31	–2.62	0.012
Session 1: Negative	11.70	1.84	11.43	2.03	0.53	0.596
Session 2: Negative	12.33	2.22	11.53	2.40	1.34	0.186

*^a^Education (in years) was estimated for 24 YA and 2 OA as they did not provide exact data, according to the following guideline: A levels = 12 years; Bachelor’s degree = 15 years; Master’s degree = 17 years; PhD = 20 years.*

*^b^FPQ scores are based on 29 OA.*

*^c^PVAQ scores from the French version (n = 3) were transformed from a 5-point to a 6-point Likert scale.*

*^d^We used boxplots to examine TMT, Stroop and flanker reaction time measures for extreme outliers. This resulted in the removal of one OA for the Stroop task; and one YA for the flanker task; another YA was removed from the flanker task due to an excessive number of errors in one condition (19 errors in 20 trials). Flanker effect scores are based on correct trials only.*

### Behavioral Distraction Effect

**FIGURE 2:**
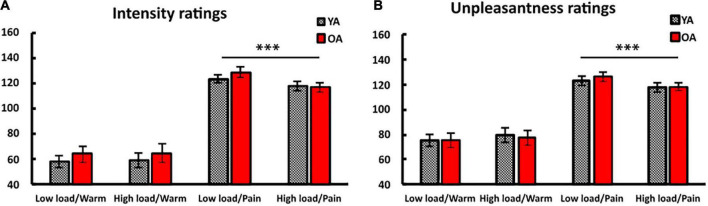
Average ratings for the four different conditions. Young (YA) and older adults (OA) rated all stimuli on **(A)** a 200-point intensity scale and **(B)** a 200-point unpleasantness scale. A rating of 100 on the intensity scale corresponds to the pain threshold (“just pain”) and a rating of 100 on the unpleasantness scale corresponds to being “neutral”. Painful stimuli were rated as significantly less intense and unpleasant when these were presented during the high load task as compared to the low load task. ***p < 0.001. Error bars represent the standard error of the mean (SEM).

Repeated measures ANOVAs with the within-subject factors *temperature level* (warm vs. painful) and *task difficulty* (low load vs. high load), and the between-subject factor *age group* (YA vs. OA) revealed significant main effects for *temperature level* on intensity ratings [*F*(1,58) = 217.31, *p* < 0.001, $\eta_{\text{p}}^{2}$ = 0.789], and unpleasantness ratings [*F*(1,58) = 121.75, *p* < 0.001, $\eta_{\text{p}}^{2}$ = 0.677]. *Task difficulty* resulted in significantly different intensity ratings [*F*(1,58) = 12.31, *p* = 0.001, $\eta_{\text{p}}^{2}$ = 0.175], but not unpleasantness ratings [*F*(1,58) = 3.77, *p* = 0.057, *$\eta_{\text{p}}^{2}$* = 0.061]. Importantly, we observed a significant interaction between *temperature level* and *task difficulty* for intensity and unpleasantness ratings [*F*(1,58) = 31.54, *p* < 0.001, $\eta_{\text{p}}^{2}$ = 0.352, and *F*(1,58) = 44.87, *p* < 0.001, $\eta_{\text{p}}^{2}$ = 0.436, see Figure 2].

Bonferroni corrected paired sample *t*-tests (critical *p* = 0.013, *k* = 4) revealed that all participants rated painful stimuli significantly lower in intensity and unpleasantness when these were presented during the high load task as compared to the low load task [*t*(59) = 5.95, *p* < 0.001 and *t*(59) = 5.39, *p* < 0.001, respectively]. Differences in intensity ratings for warm stimuli did not reach significance (*p* = 0.483) but unpleasantness ratings were significantly different (*p* = 0.009). This indicates a robust distraction effect for painful stimuli across groups. We found no significant interaction for *task difficulty* or *temperature level* with *age group* (all *p*s > 0.138), indicating that both age groups benefited to an equal degree from the high load task in terms of distraction from pain. This was also evidenced by separate repeated measures ANOVAs for young and older adults that revealed significant interactions between *temperature level* and *task difficulty* for both age groups for intensity [YA: *F*(1,29) = 8.04, *p* = 0.008, $\eta_{\text{p}}^{2}$ = 0.217; OA: *F*(1,29) = 26.77, *p* < 0.001, $\eta_{\text{p}}^{2}$ = 0.480] and unpleasantness ratings [YA: *F*(1,29) = 20.04, *p* < 0.001, $\eta_{\text{p}}^{2}$ = 0.409; OA: *F*(1,29) = 25.03, *p* < 0.001, $\eta_{\text{p}}^{2}$ = 0.463].

Note that adding the normalized (and mean-centered) gray matter volume as a covariate to these repeated measures ANOVAs did have no influence on the interaction between *temperature level* and *task difficulty* [intensity ratings: *F*(1,58) = 31.54, *p* < 0.001, $\eta_{\text{p}}^{2}$ = 0.352; unpleasantness ratings*: F*(1,58) = 44.87, *p* < 0.001, $\eta_{\text{p}}^{2}$ = 0.436].

A table with correlations between the behavioral distraction effect size and pain-related cognitions as well as executive functions can be found in the Supplementary Table 4.

### Neural Pain Response

**FIGURE 3:**
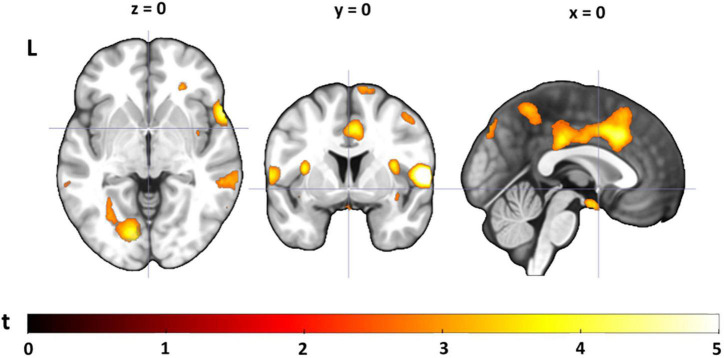
Pain-related neural activation. Painful compared to warm stimuli (pain > warm) collapsed across task conditions and groups [visualized at a threshold of *p*(unc) = 0.005, *k* ≥ 10; see also Supplementary Table 5].

To assess the neural pain response, we contrasted neural activation during the painful condition with the warm condition, collapsed across task difficulty conditions and groups (see Figure 3 and Supplementary Table 5). The contrast yielded a network of activations, including the right insula (extending into the right supramarginal gyrus), the right postcentral gyrus (primary somatosensory cortex) and left supramarginal gyrus (all surviving cluster-level FDR correction). Contrasting pain-related activations (*pain* > *warm*) for YA with OA yielded a small cluster in the right superior parietal lobe and the opposite contrast (OA > YA) revealed some small clusters in areas that are typically not associated with pain processing (see Supplementary Table 5). The clusters from these group comparisons did not survive cluster-level FDR correction. Pain-related activations for each age group separately can be found in Supplementary Tables 6, 7.

### Neural Distraction Effect

**FIGURE 4:**
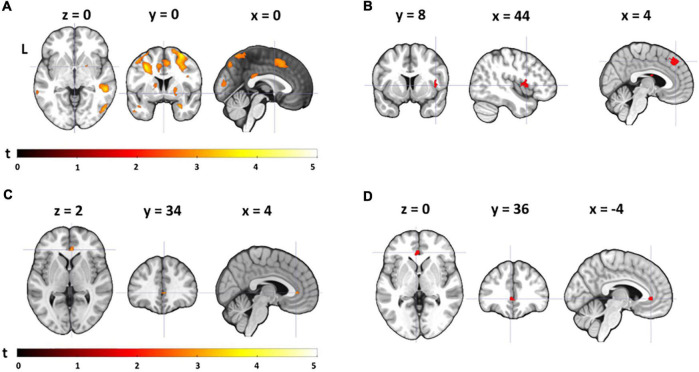
Neural distraction effect and mechanism. **(A)** Neural distraction effect (i.e., regions showing less activation during the high-load than during the low-load task) across groups (see also Table 2); **(B)** neural distraction effect for YA > OA; **(C)** neural distraction mechanism (i.e., regions showing more activation during the high-load than during the low-load task) across groups; **(D)** neural distraction mechanism for OA > YA. All contrasts are visualized at *p*(unc) = 0.005, *k* ≥ 10. Note that the color of the clusters in **(B,D)** is not indicative of the *t*-value.

We investigated the neural distraction effect by comparing the neural pain response (*pain* > *warm*) for the low load task with the high load task (*low load* > *high load*) across both groups. Exploratory analyses yielded reduced pain-related activation in the high load compared to the low load condition in the right superior and left inferior parietal lobe, the right postcentral gyrus, the right superior frontal and the left precentral gyrus, extending into the middle frontal gyrus (all surviving cluster-level FDR correction). We also found several smaller clusters in the right mid cingulate cortex (MCC) and left insula, and several frontal regions (see Figure 4A; none surviving cluster-level FDR correction). When comparing the neural distraction effect between groups, YA showed a stronger reduction in activation during the high load relative to the low load task in the right superior medial gyrus, the right insula, the right MCC, the right caudate nucleus and left supramarginal gyrus (see Table 2 and Figure 4B). None of these survived cluster-level FDR correction. The opposite contrast (OA > YA) resulted in a cluster in the right middle occipital lobe, not surviving cluster-level FDR correction (see Table 2).

**TABLE 2:** Neural distraction effect.

Brain region		MNI coordinates			Cluster			
		*x*	*y*	*z*	*p*(FDR-corr)	*k*	*T*	*Z*
***Across groups***								
Superior parietal lobule	R	38	–52	58	0.00	3027	4.81	4.66
	R	18	–60	66			3.77	3.69
Postcentral gyrus	R	44	–40	60			3.64	3.56
Inferior temporal gyrus	R	52	–64	–10	0.60	165	4.20	4.10
	R	44	–74	–6			3.32	3.27
Superior frontal gyrus	R	32	2	62	0.03	765	4.09	4.00
		26	2	50			3.69	3.62
		30	6	40			3.69	3.61
Precentral gyrus	L	–30	4	44	0.03	797	4.09	3.99
Middle frontal gyrus	L	–32	10	38			3.65	3.57
		–22	8	28			3.62	3.55
Inferior parietal lobule	L	–46	–48	54	0.01	1092	4.06	3.97
	L	–58	–46	42			4.04	3.94
Superior parietal lobule	L	–32	–56	60			3.49	3.43
Fusiform gyrus	L	–40	–66	–16	0.46	217	4.06	3.96
	L	–44	–58	–18			3.58	3.51
IFG p. orbitalis	R	28	22	–20	0.24	366	3.92	3.83
Superior orbital gyrus	R	18	38	–18			3.74	3.66
	R	20	46	–16			3.63	3.56
Middle temporal gyrus	R	54	–36	–4	0.37	272	3.84	3.76
	R	54	–28	–8			3.69	3.61
Superior temporal gyrus	L	–60	–50	16	0.93	65	3.74	3.66
Posterior medial frontal gyrus	R	10	8	48	0.06	623	3.61	3.54
	L	–8	6	44			3.38	3.32
	R	4	16	48			3.16	3.11
		–12	–20	–16	0.93	60	3.58	3.52
Middle frontal gyrus	L	–36	34	42	0.60	167	3.53	3.47
	L	–44	26	40			2.99	2.95
	L	–32	44	34			2.69	2.65
Inferior temporal gyrus	L	–44	–10	–32	0.24	349	3.53	3.47
	L	–52	–2	–34			3.41	3.35
		–40	–4	–26			3.41	3.35
Calcarine gyrus	R	20	–82	4	0.46	217	3.37	3.31
	R	4	–86	14			3.01	2.97
	R	10	–90	2			3.01	2.97
Middle frontal gyrus	R	36	36	42	0.93	87	3.34	3.29
	R	44	36	32			2.89	2.85
Inferior temporal gyrus	R	52	–24	–24	0.93	25	3.28	3.23
Parahippocampal gyrus	R	34	–26	–20	0.93	23	3.25	3.19
Olfactory cortex	L	–14	10	–16	0.93	20	3.17	3.13
Middle orbital gyrus	L	–22	42	–18	0.93	23	3.11	3.07
	L	–20	34	–20			2.97	2.93
		36	–42	32	0.93	56	3.10	3.05
		34	–50	32			2.71	2.68
Middle occipital gyrus	R	36	–76	38	0.93	55	3.01	2.97
Middle temporal gyrus	L	–64	–40	–2	0.93	18	2.96	2.92
MCC	R	6	–30	36	0.93	71	2.92	2.88
		2	–28	28			2.91	2.87
		–2	–36	22			2.75	2.71
Insula	L	–38	20	4	0.93	17	2.87	2.83
Postcentral gyrus	R	24	–38	74	0.93	20	2.85	2.82
***YA* > *OA***								
Superior medial gyrus	R	4	38	44	0.89	160	3.39	3.33
	R	8	36	54			2.85	2.82
	R	6	26	50			2.67	2.64
		6	–6	20	0.93	64	3.29	3.24
Caudate nucleus	R	14	0	22			2.81	2.78
		–14	–4	36	0.93	26	3.22	3.17
Insula	R	44	8	4	0.93	103	3.13	3.09
		34	14	–2			2.96	2.92
Insula	R	44	0	8			2.93	2.89
MCC	R	12	–14	42	0.93	15	3.02	2.98
Supramarginal gyrus	L	–54	–24	36	0.93	11	2.72	2.69
***OA* > *YA***								
Mid occipital lobe	R	34	–74	8	0.93	78	3.30	3.25

*Brain regions showing reduced activation in response to painful stimuli during the high load task when compared to the low load task [contrast: (pain > warm) _low load_ > (pain > warm) _high load_] at p(unc) = 0.005 and k ≥ 10 and cluster correction FDR p-levels indicated separately.*

Adding pain catastrophizing (total PCS score), fear of pain (total FPQ-III score), medication intake or self-reported positive affect (PANAS subscale) on the day of the test session as covariates to these contrasts did not change the results (see Supplementary Figures 1–3). Results for the neural distraction effect for each age group separately can be found in Supplementary Tables 8, 9.

### Neural Distraction Mechanism

To investigate brain regions that play a role in generating the distraction effect (i.e., that are involved in descending pain control), we also explored the *pain* > *warm* contrast for the high load > low load task condition. When looking across groups, we found a cluster in the perigenual anterior cingulate cortex (ACC) (not surviving cluster-level FDR correction) (peak MNI coordinates: *x* = 4, *y* = 34, *z* = 2; *k* = 12, *Z* = 2.99; Figure 4C). Group comparisons showed that this cluster was significantly more activated in OA than in YA (peak MNI coordinates: *x* = −4, *y* = 36, *z* = 0; Figure 4D) whereas the opposite contrast (YA > OA) yielded no clusters.

### Correlations Between Neural Effects and Executive Functions

**FIGURE 5:**
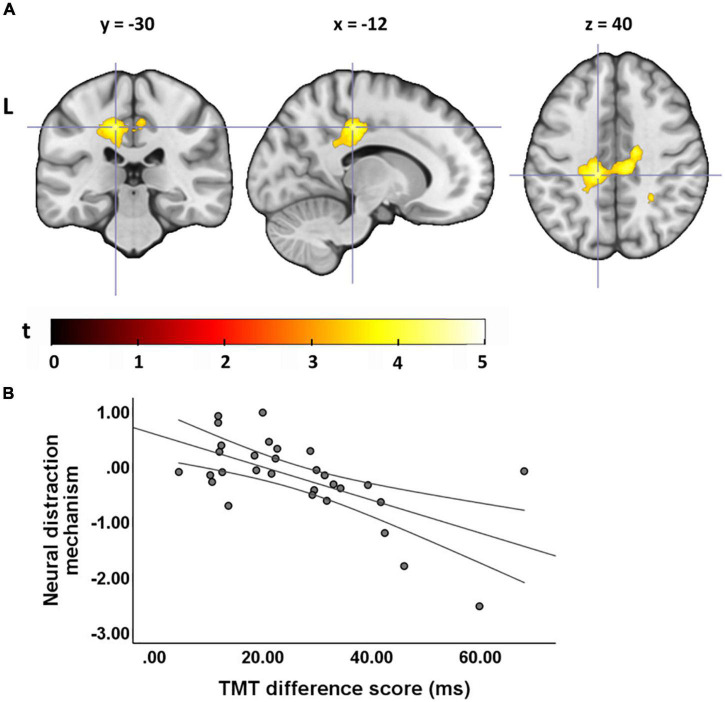
Correlations between executive functions and the neural distraction mechanism in young adults. **(A)** Regions showing a negative correlation between the TMT difference score and the neural distraction mechanism [(pain > warm) _high load_ > (pain > warm) _low load_] in young adults [visualized at *p*(unc) = 0.001, *k* ≥ 20; see also Table 3]. **(B)** Parameter estimates extracted from the cluster maximum in the mid cingulate gryus (position of cross hairs in **A**) for the neural distraction mechanism contrast. A smaller TMT difference score indicates better executive functions.

**FIGURE 6:**
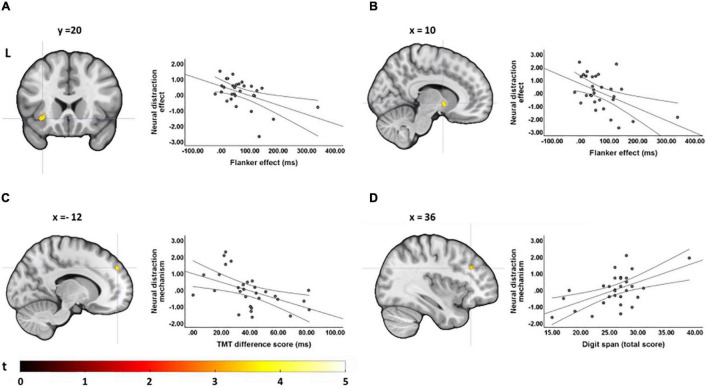
Correlations between executive functions and the neural distraction effect and mechanism in older adults. **(A)** A smaller flanker effect (i.e., better interference control abilities) was associated with a greater neural distraction effect [(pain > warm) _low load_ > (pain > warm) _high load_] in the left insula and **(B)** the right thalamus (see also Table 4); **(C)** smaller difference scores in the TMT (i.e., better executive functions) were associated with a greater neural distraction mechanism [(pain > warm) _high load_ > (pain > warm) _low load_] in the left superior frontal gyrus (see also Table 3); **(D)** digit span (i.e., working memory ability) correlated positively with the neural distraction mechanism in the left inferior frontal gyrus and right middle frontal gyrus. [All activation maps are visualized at *p*(unc) = 0.001, *k* ≥ 20].

For young adults, we found no correlation between EFs and the neural distraction effect, but for the distraction mechanism we found that a smaller flanker effect and TMT difference score (i.e., better interference control and general executive functions) were related to an increased recruitment of areas in the cingulate gyrus and supramarginal gyrus (Table 3 and Figure 5). One cluster in the left MCC, which correlated with the TMT difference score, survived cluster-level FDR correction. In older adults, we found a negative correlation between the flanker and Stroop effect and the neural distraction effect, such that better inhibitory control abilities were related to a stronger distraction-related reduction of activation in the left insula, right thalamus, left postcentral gyrus and left precuneus (Table 4 and Figures 6A,B). The flanker effect and TMT difference score were also negatively associated with the neural distraction mechanism in the left middle temporal and inferior temporal gyri as well as the left superior frontal gyrus, and a longer digit span (i.e., better working memory abilities) was associated with a larger neural distraction mechanism in the left inferior and right middle frontal gyri (Table 3 and Figures 6C,D). None of the correlations in the OA survived cluster-level FDR correction. Correlations between the neural and behavioral distraction effect can be found in the Supplementary Table 10.

**TABLE 3:** Correlations between the neural distraction mechanism and executive functions.

Covariate	Direction of corr.	Anatomical label		MNI coordinates			Cluster			
				*x*	*y*	*z*	*p*(FDR-corr)	*k*	*T*	*Z*
***YA***										
Flanker effect	neg.		R	24	24	18	0.88	26	3.73	3.50
		MCC	R	14	–30	44	0.88	26	3.65	3.43
TMT difference	neg.	MCC	L	–12	–30	40	0.00	911	4.59	4.21
			L	–14	–22	40			4.47	4.12
		Cingulate gyrus	R	18	–12	44			4.36	4.03
		Inferior parietal lobule	R	58	–50	38	0.88	34	4.09	3.81
		Angular gyrus	R	60	–50	30			3.56	3.36
		Intraparietal sulcus	R	26	–46	36	0.83	60	3.94	3.69
***OA***										
Flanker effect	neg.	Middle temporal gyrus	L	–60	–20	-18	0.88	47	3.97	3.70
		Inferior temporal gyrus	L	–54	–20	-26			3.50	3.31
TMT difference	neg.	Superior frontal gyrus	L	–12	50	36	0.88	29	3.98	3.72
Digit span (total)	pos.	Inferior frontal gyrus	L	–50	38	8	0.88	38	4.18	3.88
			R	36	30	28	0.88	67	3.72	3.50
		Middle frontal gyrus	R	40	38	28			3.63	3.43
			R	38	50	16			3.59	3.39

*Brain regions showing a correlation between the neural distraction mechanism and executive functions in young (YA) and older (OA) adults at p(unc) = 0.001 and k ≥ 20 and cluster correction FDR p-levels indicated separately.*

**TABLE 4:** Correlations between the neural distraction effect and executive functions in OA.

Covariate	Direction of corr.	Anatomical label		MNI coordinates			Cluster			
				*x*	*y*	*z*	*p*(FDR-corr)	*k*	*T*	*Z*
Flanker effect	neg.	Insula	L	–30	20	-8	0.83	64	3.95	3.68
		Thalamus	R	10	2	-2	0.83	30	3.85	3.60
Stroop effect	neg.	Postcentral gyrus	L	–26	–50	68	0.24	133	4.39	4.05
				–14	–56	64			4.00	3.73
		Precuneus	L	–22	–56	42	0.24	124	4.21	3.90
				–16	–52	48			3.42	3.24

*Brain regions showing a correlation between the neural distraction effect and executive functions in older adults at p(unc) = 0.001 and k ≥ 20.*

### Gray Matter Volume and Distraction Effect

**FIGURE 7:**
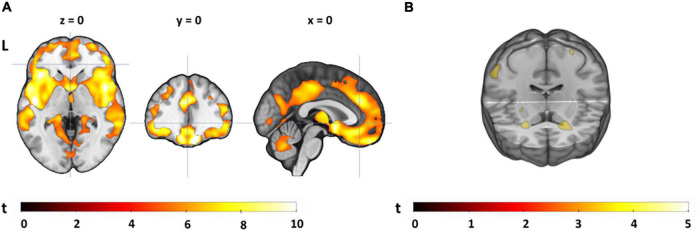
Results from the gray matter volume analyses. **(A)** Young adults > older adults contrast for the GM images (visualized at a FWE-corrected *p* = 0.05, *k* ≥ 20; see also Supplementary Table 11). **(B)** Regions showing a positive correlation between the behavioral distraction effect size (on the unpleasantness scale) and GM volume in older adults [visualized at *p*(unc) = 0.001, *k* ≥ 20; see also Table 5].

A group comparison showed widespread age-related atrophy in GM volume in OA compared to YA, especially apparent in frontal, temporal and parietal regions, and with the largest group differences in the straight gyrus, and in the angular gyrus (see Supplementary Table 11). Exploratory analyses adding VAS pain ratings to GM volume maps revealed a positive correlation between the distraction effect on the intensity scale and GM volume in a small cluster in the right middle temporal gyrus for YA; for the distraction effect on the unpleasantness scale, we found positive correlations with GM volume in several clusters in the right temporal gyri for YA, and in two larger clusters in the right supramarginal gyrus and the right and left amygdalae for OA (Table 5 and Figure 7). None of these survived cluster-level FDR-correction. Note that adding GM volume (controlled for total intracranial volume) as covariate to group comparisons of the neural distraction effect and mechanism did not change the results (see Supplementary Tables 12, 13).

**TABLE 5:** Correlations between GM volume and the behavioral distraction effect.

Covariate	Direction of corr.	Anatomical label		MNI coordinates			Cluster			
				*x*	*y*	*z*	*p*(FDR-corr)	*k*	*T*	*Z*
***DE-I***										
YA	pos.	Middle temporal gyrus	R	54	–51	14	0.86	72	3.70	3.48
OA	pos.	–	–	−	−	−	–	–	–	–
***DE-U***										
YA	pos.	Precuneus	L	–9	–44	45	0.95	51	3.90	3.64
		Superior temporal gyrus	R	65	–6	5	0.95	30	3.54	3.35
		Cerebellar vermis (4/5)	R	6	–50	–12	0.95	31	3.50	3.31
OA	pos.	Supramarginal gyrus	R	57	–42	24	0.77	175	3.91	3.65
		Supramarginal gyrus	R	51	–42	36			3.61	3.40
		Amygdala	R	24	–2	–14	0.78	71	3.90	3.65
		Amygdala	L	–18	–2	–14	0.77	146	3.82	3.58
		Middle occipital gyrus	L	–29	–72	35	0.78	49	3.50	3.31

*DE-I, behavioral distraction effect on intensity scale; DE-U, behavioral distraction effect on unpleasantness scale. Gray matter (GM) volume was corrected for the total intracranial volume for each participant. Contrast at p(unc) = 0.001 and k ≥ 20 and cluster correction FDR p-levels indicated separately.*

## Discussion

The aim of the present study was to examine age-related changes in cognitive distraction from pain on the behavioral and neural level, and whether these changes would be mediated by differences in prefrontal functioning, as indexed by executive functions and gray matter volume.

Although the older participants showed a significant deterioration in EFs and reduced GM volume throughout the cortex when compared to our younger participants, both groups benefited to an equal degree from the distractive properties of the high load task, when looking at their pain ratings. This finding is in line with a recent study assessing age-related changes in the electrophysiological correlates of distraction from pain with a similar paradigm (González-Roldán et al., 2020a). One potential explanation for the preserved behavioral distraction effect is that older adults may have compensated for the decline in EFs and GM volume with an increased activation of brain structures involved in the cognitive modulation of pain, such as the PFC and ACC. A similar hypothesis has been offered for pain distraction studies with chronic pain patients (who show a heightened focus on pain stimuli), which observed no differences between patients and healthy controls in the behavioral distraction effect size (Nouwen et al., 2006; Schreiber et al., 2014; Stankewitz et al., 2018), presumably because of compensatory neural mechanisms (Stankewitz et al., 2018). Thus, age-related changes in distraction from pain may only become apparent either if the intensity of the nociceptive stimulus surpasses the capacity of the compensatory mechanisms to modulate the pain intensity, or if age-related decline in cortical plasticity due to atrophy has progressed too far, impeding the effective engagement of these compensatory descending pain control mechanisms. Our results indeed suggest that OA may have succeeded in employing a compensatory mechanism during the high-load task (i.e., in the distraction condition), as they showed increased activation in the ACC, compared to YA. This will be discussed further below.

Engaging in a cognitively demanding task during painful stimulation resulted in a reduction of activity in several large clusters in frontal and parietal regions (including the right primary somatosensory cortex) across both groups that have been reported in previous neuroimaging studies on distraction from pain (Petrovic et al., 2000; Valet et al., 2004; Wiech et al., 2005; Seminowicz and Davis, 2007). Exploratory analyses showed that this effect was significantly smaller in several regions in OA (including the right insula, right MCC and left supramarginal gyrus). However, OA with better cognitive inhibition abilities, i.e., a smaller flanker and Stroop effect, showed a larger neural distraction effect in the left insula, right thalamus, the left postcentral gyrus (primary somatosensory cortex) and left precuneus. This is in line with a growing body of studies suggesting a link between cognitive inhibition abilities and pain sensitivity and modulation (Oosterman et al., 2010, 2016; Marouf et al., 2014; Zhou et al., 2015b; Bjekić et al., 2018; Bunk et al., 2020). To our knowledge, this study is the first to link cognitive inhibition abilities directly to the efficacy of attentional pain modulation in the brain (in older adults). We found, however, no such association between cognitive inhibition abilities and the neural distraction effect in YA; this is likely due to YA showing less variation in their EFs whereas OA showed decline in performance to various degrees.

Both age groups showed a significant increase in neural activation during the high load vs. low load task in the perigenual ACC (as revealed with a liberal threshold). This cluster was significantly more active in OA than in YA. Covariate analyses further revealed that OA with better executive functions (flanker test, TMT, digit span) showed stronger recruitment of several frontal and temporal regions during distraction from pain (only at a liberal threshold), whereas YA with better EFs (flanker, TMT) showed stronger activation in the MCC during the high load relative to the low load task.

While the stronger recruitment of prefrontal regions in older adults with better executive functions is in line with our hypothesis that the PFC plays a pivotal role in initiating cognitive pain modulation (Wiech et al., 2008; Knudsen et al., 2011), increased activation in temporal areas has been less systematically reported in the context of pain modulation. It is, however, noteworthy that increased activation in the temporal lobe (at a similar location as in our study) has been previously found in a study that compared externally controlled with self-controlled painful stimuli, suggesting that this specific brain area may play a role in the perceived control of pain (Wiech et al., 2006). Given that increased activity in the temporal lobe in our study was associated with better cognitive inhibition abilities in older adults, it could be speculated that perceived pain control in older adults with better inhibition abilities was different from older adults with worse inhibition abilities. Future research could explicitly address this question by assessing the role of cognitive inhibition abilities in pain paradigms on pain controllability.

The ACC has been identified as a key structure in the attentional modulation of pain in a series of distraction studies (Frankenstein et al., 2001; Bantick et al., 2002; Valet et al., 2004; Seminowicz and Davis, 2007) and has also been implicated in descending pain control (Wiech et al., 2008; Sprenger et al., 2011) as well as more general affect regulation (Shackman et al., 2011; Stevens, 2011). Interestingly, the ACC constitutes part of an emotion-related network that is relatively spared from age-related decline in comparison to cognitive networks (Nashiro et al., 2017). Taken together, this suggests that OA rely more strongly on the relatively preserved emotion circuit structures for pain modulation, possibly to compensate for a decline in function and integrity of frontal regions (as discussed above). In addition, our results suggest that OA with better EFs may still be able to recruit resources in frontal regions for pain modulation. Further studies are necessary to investigate the role of emotion regulation abilities and how these relate to preserved cognitive pain modulation in healthy aging. Interestingly, we observed that YA who performed better on the TMT showed an increased recruitment of the MCC during the high load task. Similar to the perigenual ACC, this region receives many projections from the pain-related thalamic nuclei; however, in contrast to the ACC, the MCC has extensive connections with prefrontal and motor-related areas of the cortex (Stevens, 2011). Although the segregation of the cingulate cortex into an area specialized for affective processes (ACC) and an area specialized for cognitive processes (MCC) is equivocal (Shackman et al., 2011), the observed association between better executive functions in YA and an increased recruitment of the MCC during the high load task could point to a stronger involvement of PFC regions during distraction in YA relative to OA.

Our data also suggest that age-related changes in the gray matter volume in the bilateral amygdalae and right supramarginal gyrus modulate the distraction effect size on the behavioral level. The amygdala has been implicated in the affective aspects of pain processing (Neugebauer et al., 2009) and may influence descending pain pathways via medullary mechanisms (Bannister, 2019) while the supramarginal gyrus forms part of the somatosensory association cortex and its GM volume has been associated with the preservation of emotion recognition ability in older adults (Wada et al., 2021). Both structures were also found to show reduced GM volume in patients with chronic pain (Burgmer et al., 2009; Coppieters, 2017). Altogether, these results support the notion that age-related changes in the brain primarily influence the affective aspects of pain perception (Neugebauer et al., 2009; González-Roldán et al., 2020a; Terrasa et al., 2021).

We found no indication for a mediating role of total GM volume in age-related differences in behavioral or neural distraction effect measures. An explanation for this lack of an association could be cognitive reserve or compensatory mechanisms (Cabeza et al., 2018). Possibly, these mechanisms were not sufficient to compensate for GM atrophy in the brain structures for which an association between GM volume and the behavioral distraction effect was observed.

### Limitations

One limitation of our study is that the OA who volunteered to participate were generally characterized by high positive affect (and motivation as indicated by the post-experimental questionnaire) and low negative-pain related cognitions (as also observed in other studies, see e.g., González-Roldán et al., 2020b; Zhou et al., 2020). They were also free from chronic pain, relatively highly educated and in good health, meeting all criteria required for an MRI scan (i.e., no metal implants, insulin pumps etc.) (Ganguli et al., 2015). Moreover, most Luxembourgish citizens grow up in a trilingual environment (Luxembourgish, German, French), which has been shown to have beneficial effects for cognitive aging (Bialystok et al., 2014). Therefore, our results may not be easily generalizable to the overall aging population. We also observed that the magnitude of the behavioral distraction effect in the present study was significantly smaller than the magnitude that we observed in a previous study run in our lab, using the identical paradigm (Rischer et al., 2020). This difference could be explained by the requirements and contextual factors associated with an MRI scan (i.e., constant noise, requirement not to move, constrained space), which may have made it more difficult for participants to focus on the working memory task. In addition, instead of comparing the distractive task to a no task condition or instructing participants to focus on the stimulation as e.g., done in Petrovic et al. (2000) and Valet et al. (2004), both the distractive (high load) task and the control (low load) task required the continuous allocation of attentional resources. Together with the (unintended) selection and recruitment bias, this factor may have contributed to our relatively small behavioral and neural distraction effect (e.g., most neural effects only appeared when using a more liberal threshold). Another reason for the relatively small neural effects (apart from the neural pain response) could have been that complex cognitive and affective processes, such as cognitive pain modulation, are associated with a relatively larger intra- and inter-person variability in neural activation patterns than e.g., pure sensory or motor phenomena (Lieberman and Cunningham, 2009). This is also illustrated by the fact that additional analyses of the neural distraction effect and mechanism using specific regions of interest [using the AAL atlas from Tzourio-Mazoyer et al. (2002) as implemented in the WFU-Pickatlas] did not survive FDR-correction despite a restricted search volume. Tables with the clusters from these analyses (and a specification of the anatomical regions used) can be found in the Supplementary Tables 14, 15.

Future studies could specifically compare distraction from pain in healthy aging adults with adults facing cognitive impairments e.g., due to dementia, or pre-select study participants depending on their cognitive status (Zhou et al., 2020) and thereby increase the heterogeneity of the sample characteristics. Given that executive functions, specifically cognitive inhibition abilities, have been associated with protective effects against pain-induced interference on task performance (Verhoeven et al., 2011), it would also be interesting to assess the role of executive functions on distraction task performance in future studies. A further important focus would be to elucidate the role of an (age-related) decline in cognitive pain modulation in the development of chronic pain by comparing chronic pain patients and healthy matched controls in the advanced stages of life.

Taken together, our findings demonstrate clear age-related changes in the top–down attentional modulation of pain, and suggest that the preservation of EFs, specifically inhibitory control abilities, and GM volume in the amygdalae and supramarginal gyrus in advanced age, may protect from a decline in efficacy of cognitive pain coping strategies. Cognitive training that aims at preserving or improving EFs may be a promising avenue to prevent older adults from developing, or to support them in coping with, chronic pain conditions.

## Data Availability Statement

The data supporting the findings of this study are available from the corresponding author upon reasonable request. They are not publicly available because of ethical restrictions.

## Ethics Statement

The study was reviewed and approved by Ethics Review Panel at the University of Luxembourg and the Comité National d’Ethique de Recherche Luxembourg (CNER). All participants provided their written informed consent to participate in this study.

## Author Contributions

MM conceptualized and designed the study together with AG-R. MM and KR implemented the study and collected the data. KR collected the majority of the data. KR processed and analyzed the data under supervision of MM. KR provided the first draft of the manuscript which was subsequently critically revised by all authors. All authors contributed to the article and approved the submitted version.

## Conflict of Interest

The authors declare that the research was conducted in the absence of any commercial or financial relationships that could be construed as a potential conflict of interest.

## Publisher’s Note

All claims expressed in this article are solely those of the authors and do not necessarily represent those of their affiliated organizations, or those of the publisher, the editors and the reviewers. Any product that may be evaluated in this article, or claim that may be made by its manufacturer, is not guaranteed or endorsed by the publisher.
