# Supplementary material for: Better Executive Functions Are Associated With More Efficient Cognitive Pain Modulation in Older Adults: An fMRI Study
Source: Front Aging Neurosci. 2022 Jul 7;14:828742. doi: 10.3389/fnagi.2022.828742 (PMC9302198; doi:10.3389/fnagi.2022.828742)
Supplement: Supplementary file 1 [file Table_1.DOCX]

**Table S1: Distraction paradigm settings.**

|  | Young adults | |  | Older adults | |  |  |
| --- | --- | --- | --- | --- | --- | --- | --- |
|  | *Mean* | *SD* |  | *Mean* | *SD* | *t-statistic*  *df = 58* | *p-value* |
| Warm temperature (°C) | 43.58 | 1.19 |  | 42.44 | 1.67 | 3.02 | .004 |
| Painful temperature (°C) | 46.90 | 0.68 |  | 46.84 | 0.94 | 0.26 | .798 |
| Average task speed  (ms between letters) | 890.30 | 435.88 |  | 1558.73 | 587.70 | -5.00 | < .001 |
