# Supplementary material for: Better Executive Functions Are Associated With More Efficient Cognitive Pain Modulation in Older Adults: An fMRI Study
Source: Front Aging Neurosci. 2022 Jul 7;14:828742. doi: 10.3389/fnagi.2022.828742 (PMC9302198; doi:10.3389/fnagi.2022.828742)
Supplement: Supplementary file 2 [file Table_2.DOCX]

**Table S2: Medication intake.**

|  | Young adults  (n = 30) | Older adults  (n = 30) |
| --- | --- | --- |
| *Medication* |  |  |
| Anxiolytic | 0 | 0 |
| Antidepressant^a^ | 0 | 4 |
| Anti-inflammatory | 0 | 0 |
| Cholesterol | 0 | 6 |
| Hypertension | 0 | 7 |
| Hypoglycemic | 0 | 3 |
| Other | 3 | 9 |
| Hormones^b^ | 5 | 1 |

^a^ Participants reporting to take antidepressants were long-term users and did not suffer from an acute depressive episode; ^b^ All participants who reported to take hormones were female.
