# Supplementary material for: Better Executive Functions Are Associated With More Efficient Cognitive Pain Modulation in Older Adults: An fMRI Study
Source: Front Aging Neurosci. 2022 Jul 7;14:828742. doi: 10.3389/fnagi.2022.828742 (PMC9302198; doi:10.3389/fnagi.2022.828742)
Supplement: Supplementary file 3 [file Table_3.DOCX]

**Table S3 : Post-experimental questionnaire.**

|  | Young adults | |  | Older adults | |  |  |
| --- | --- | --- | --- | --- | --- | --- | --- |
|  | *Mean* | *SD* |  | *Mean* | *SD* | *t-statistic* | *p* |
| Attention to thermal stimuli | 5.20 | 2.51 |  | 5.67 | 2.41 | -.734 | 0.466 |
| *0-back task* |  |  |  |  |  |  |  |
| Perceived difficulty | 1.27 | 1.23 |  | 1.27 | 1.29 | 0.00 | 1.000 |
| Ambition to perform well ^a^ | 9.07 | 0.94 |  | 7.87 | 2.57 | 2.40 | 0.022 |
| Task-induced stress | 2.13 | 2.27 |  | 1.53 | 1.89 | 1.11 | 0.270 |
| Perceived distraction from pain | 5.23 | 2.90 |  | 3.87 | 2.92 | 1.82 | 0.074 |
| *2-back task* |  |  |  |  |  |  |  |
| Perceived difficulty | 6.13 | 1.87 |  | 6.77 | 1.76 | -1.35 | 0.182 |
| Ambition to perform well | 8.43 | 1.04 |  | 7.63 | 2.34 | 1.71 | 0.095 |
| Task-induced stress | 5.10 | 2.64 |  | 5.50 | 2.57 | -.594 | 0.555 |
| Perceived distraction from pain | 6.67 | 2.77 |  | 5.63 | 2.77 | 1.44 | 0.154 |

^a^ Older adults (OA) rated their ambition to perform well in the low load condition lower than YA. However further inspection of the data revealed that only 4 OA rated their ambition with 3 points or less (whereas all other OA rated their ambition with 7 points or more); excluding these 4 participants, nullifies the observed difference.
