# Supplementary material for: Better Executive Functions Are Associated With More Efficient Cognitive Pain Modulation in Older Adults: An fMRI Study
Source: Front Aging Neurosci. 2022 Jul 7;14:828742. doi: 10.3389/fnagi.2022.828742 (PMC9302198; doi:10.3389/fnagi.2022.828742)
Supplement: Supplementary file 4 [file Table_4.DOCX]

**Table S4: Correlations between the behavioral distraction effect and pain-related cognitions as well as executive functions.**

|  |  | | FPQ-III (total) | PCS (total) | Digit span (total) | Flanker effect | Stroop effect | TMT difference score |
| --- | --- | --- | --- | --- | --- | --- | --- | --- |
| Younger adults |  | | *n* = 30 | *n* = 30 | *n* = 30 | *n* = 28 | *n* = 30 | *n* = 30 |
| DE-I ^a^ | *r* | | -0.005 | -0.143 | 0.129 | -0.077 | .501^c^ | -0.104 |
|  | *p* | | 0.980 | 0.451 | 0.495 | 0.698 | 0.005 | 0.583 |
|  | LLCI | | -0.433 | -0.447 | -0.260 | -0.417 | 0.002 | -0.370 |
|  | ULCI | | 0.407 | 0.191 | 0.479 | 0.314 | 0.748 | 0.150 |
| DE-U ^b^ | *r* | | 0.117 | -0.063 | 0.206 | 0.004 | 0.108 | -0.311 |
|  | *p* | | 0.538 | 0.743 | 0.274 | 0.984 | 0.569 | 0.094 |
|  | LLCI | | -0.326 | -0.392 | -0.257 | -0.331 | -0.246 | -0.541 |
|  | ULCI | | 0.435 | 0.255 | 0.588 | 0.352 | 0.482 | -0.020 |
|  |  | |  |  |  |  |  |  |
|  |  | | FPQ-III (total) | PCS (total) | Digit span (total) | Flanker effect | Stroop effect | TMT difference score |
| Older adults |  | | *n* = 29 | *n* = 30 | *n* = 30 | *n* = 30 | *n* = 29 | *n* = 30 |
| DE-I ^a^ | | *r* | -0.062 | -0.020 | -0.155 | 0.129 | -0.177 | -0.144 |
|  | | *p* | 0.748 | 0.915 | 0.415 | 0.497 | 0.359 | 0.449 |
|  | | LLCI | -0.358 | -0.365 | -0.485 | -0.252 | -0.589 | -0.514 |
|  | | ULCI | 0.227 | 0.279 | 0.347 | 0.496 | 0.219 | 0.216 |
| DE-U ^b^ | | *r* | 0.056 | 0.118 | -0.185 | -0.111 | -0.254 | -0.031 |
|  | | *p* | 0.773 | 0.533 | 0.327 | 0.560 | 0.183 | 0.871 |
|  | | LLCI | -0.290 | -0.237 | -0.489 | -0.409 | -0.598 | -0.391 |
|  | | ULCI | 0.403 | 0.455 | 0.176 | 0.197 | 0.191 | 0.345 |

Pearson correlations between the behavioral distraction effect (DE-I and DE-U) and pain-related cognitions that showed a significant age-related difference and between executive functions for each age group. Bias-corrected and accelerated 95% confidence intervals (lower boundary: LLCI; upper boundary: ULCI) are reported using bootstrapping (1,000 samples). ^a^ DE-I: Behavioral distraction effect on the intensity scale; ^b^ Behavioral distraction effect on the unpleasantness scale; ^c^ Scatter plots showed that this correlation was driven by two outliers; removing these outliers rendered the correlation insignificant.
