# Supplementary material for: Better Executive Functions Are Associated With More Efficient Cognitive Pain Modulation in Older Adults: An fMRI Study
Source: Front Aging Neurosci. 2022 Jul 7;14:828742. doi: 10.3389/fnagi.2022.828742 (PMC9302198; doi:10.3389/fnagi.2022.828742)
Supplement: Supplementary file 5 [file Table_5.DOCX]

**Table S5: Neural pain response.**

| Anatomical labels |  | MNI coordinates | | |  | Cluster | | | |
| --- | --- | --- | --- | --- | --- | --- | --- | --- | --- |
|  |  | x | y | z |  | *p*(FDR-corr) | *k* | *T* | *Z* |
| *Pain > Warm across groups* |  |  |  |  |  |  |  |  |  |
| Insula | R | 36 | -16 | 18 |  | 0.00 | 11203 | 8.27 | 7.59 |
| Supramarginal gyrus | R | 50 | -24 | 24 |  |  |  | 8.06 | 7.42 |
| Postcentral gyrus | R | 22 | -42 | 72 |  |  |  | 7.22 | 6.75 |
| Supramarginal gyrus | L | -64 | -30 | 26 |  | 0.00 | 1694 | 6.76 | 6.36 |
| Inferior parietal lobule | L | -58 | -48 | 40 |  |  |  | 3.83 | 3.74 |
| Supramarginal gyrus | L | -56 | -36 | 36 |  |  |  | 3.73 | 3.65 |
| Superior orbital gyrus | R | 24 | 32 | -14 |  | 0.14 | 416 | 5.42 | 5.20 |
| Olfactory cortex | R | 22 | 12 | -20 |  |  |  | 4.27 | 4.16 |
| IFG p. orbitalis | R | 26 | 20 | -20 |  |  |  | 2.95 | 2.91 |
| Rolandic operculum | L | -56 | -2 | 8 |  | 0.23 | 293 | 5.02 | 4.85 |
| Superior orbital gyrus | L | -20 | 36 | -14 |  | 0.14 | 413 | 4.88 | 4.72 |
| IFG p. orbitalis | L | -22 | 16 | -20 |  |  |  | 4.29 | 4.18 |
|  |  | -22 | 24 | -12 |  |  |  | 3.61 | 3.54 |
| Calcarine gyrus | L | -10 | -84 | 2 |  | 0.00 | 1308 | 4.63 | 4.49 |
| Fusiform gyrus | L | -32 | -66 | -10 |  |  |  | 3.86 | 3.77 |
| Lingual gyrus | L | -16 | -70 | -8 |  |  |  | 3.74 | 3.66 |
| Insula | L | -34 | 6 | 10 |  | 0.61 | 144 | 4.49 | 4.36 |
| Insula | L | -32 | 22 | 8 |  |  |  | 3.28 | 3.22 |
| Insula | R | 34 | 8 | 14 |  | 0.31 | 237 | 4.28 | 4.17 |
| Insula | R | 34 | 28 | 8 |  |  |  | 3.25 | 3.20 |
|  |  | 28 | 22 | 14 |  |  |  | 2.96 | 2.92 |
|  |  | 0 | 0 | -16 |  | 0.93 | 92 | 3.63 | 3.56 |
| Cuneus | R | 8 | -82 | 38 |  | 0.14 | 382 | 3.48 | 3.42 |
| Precuneus | R | 4 | -76 | 50 |  |  |  | 3.07 | 3.03 |
| Cuneus | R | 12 | -72 | 34 |  |  |  | 3.00 | 2.96 |
| Middle occipital gyrus | L | -48 | -76 | 2 |  | 0.93 | 68 | 3.44 | 3.38 |
| Middle frontal gyrus | R | 50 | 44 | 12 |  | 0.93 | 41 | 3.41 | 3.35 |
| Inferior temporal gyrus | L | -56 | -14 | -30 |  | 0.93 | 21 | 3.16 | 3.12 |
| Lingual gyrus | R | 26 | -58 | 2 |  | 0.93 | 30 | 3.16 | 3.11 |
| Insula | L | -36 | -18 | 18 |  | 0.93 | 13 | 3.06 | 3.02 |
| Middle frontal gyrus | L | -34 | 48 | 28 |  | 0.93 | 30 | 3.06 | 3.01 |
| Middle temporal gyrus | L | -58 | -52 | 0 |  | 0.93 | 75 | 3.03 | 2.98 |
| Middle temporal gyrus | L | -62 | -42 | -2 |  |  |  | 2.86 | 2.83 |
|  |  | 38 | 0 | -2 |  | 0.93 | 20 | 2.99 | 2.95 |
|  |  | 36 | 4 | -12 |  |  |  | 2.96 | 2.92 |
| Lingual gyrus | R | 4 | -66 | 8 |  | 0.93 | 35 | 2.97 | 2.93 |
| Posterior medial frontal | R | 8 | 14 | 64 |  | 0.93 | 19 | 2.94 | 2.90 |
| *Pain > Warm for YA > OA* |  |  |  |  |  |  |  |  |  |
| Superior parietal lobule | R | 32 | -74 | 48 |  | 0.93 | 28 | 3.23 | 3.18 |
|  |  | 14 | -34 | 32 |  | 0.93 | 14 | 2.87 | 2.83 |
| *Pain > Warm for OA > YA* |  |  |  |  |  |  |  |  |  |
| Mid fusiform gyrus | L | -36 | -50 | -8 |  | 0.93 | 77 | 3.40 | 3.35 |
|  |  | -42 | -54 | 2 |  |  |  | 3.14 | 3.09 |
|  |  | 2 | 40 | 54 |  | 0.93 | 43 | 3.10 | 3.06 |
|  |  | 48 | -40 | -4 |  | 0.93 | 22 | 3.06 | 3.02 |
| Middle frontal gyrus | R | 24 | 34 | -12 |  | 0.93 | 10 | 2.88 | 2.84 |
| Superior frontal gyrus | R | 18 | -16 | 68 |  | 0.93 | 12 | 2.72 | 2.69 |

YA = young adults; OA = older adults; Brain regions showing increased activation in response to painful compared to warm stimuli at *p*(unc) = .005 and *k* ≥ 10, and cluster correction FDR p-levels indicated separately.
