# Supplementary material for: Better Executive Functions Are Associated With More Efficient Cognitive Pain Modulation in Older Adults: An fMRI Study
Source: Front Aging Neurosci. 2022 Jul 7;14:828742. doi: 10.3389/fnagi.2022.828742 (PMC9302198; doi:10.3389/fnagi.2022.828742)
Supplement: Supplementary file 6 [file Table_6.DOCX]

**Table S6: Neural pain response in young adults.**

| Anatomical label |  | MNI coordinates | | | Cluster | | | |
| --- | --- | --- | --- | --- | --- | --- | --- | --- |
|  |  | x | y | z | *p*(FDR-corr) | *k* | *T* | *Z* |
| Insula Lobe | R | 36 | -16 | 18 | 0.00 | 3940 | 6.83 | 6.42 |
| SupraMarginal Gyrus | R | 56 | -24 | 24 |  |  | 5.99 | 5.70 |
| Postcentral Gyrus | R | 34 | -34 | 66 |  |  | 4.89 | 4.73 |
| SupraMarginal Gyrus | L | -64 | -26 | 26 | 0.03 | 776 | 5.07 | 4.89 |
| Superior Temporal Gyrus | L | -56 | -32 | 18 |  |  | 4.02 | 3.93 |
| SupraMarginal Gyrus | L | -58 | -52 | 32 |  |  | 3.31 | 3.26 |
| IFG p. Orbitalis | L | -22 | 16 | -20 | 0.84 | 155 | 4.08 | 3.98 |
| IFG p. Orbitalis | L | -22 | 8 | -22 |  |  | 3.73 | 3.66 |
| IFG p. Orbitalis | L | -28 | 20 | -24 |  |  | 3.50 | 3.44 |
| Lingual Gyrus | L | -12 | -76 | -6 | 0.15 | 436 | 3.87 | 3.79 |
| Lingual Gyrus | L | -18 | -68 | -10 |  |  | 3.76 | 3.69 |
| Calcarine Gyrus | L | -8 | -84 | 2 |  |  | 3.70 | 3.62 |
| Cuneus | R | 14 | -82 | 44 | 0.59 | 217 | 3.82 | 3.73 |
| Precuneus | R | 6 | -80 | 46 |  |  | 3.46 | 3.40 |
| Precuneus | L | 0 | -76 | 50 |  |  | 3.24 | 3.19 |
| MCC | R | 4 | -16 | 38 | 0.01 | 1028 | 3.76 | 3.68 |
| MCC | R | 4 | 2 | 40 |  |  | 3.56 | 3.49 |
| MCC | L | -6 | -24 | 40 |  |  | 3.52 | 3.46 |
| IFG p. Orbitalis | R | 24 | 28 | -18 | 0.93 | 44 | 3.45 | 3.39 |
| IFG p. Orbitalis | R | 26 | 18 | -22 |  |  | 3.30 | 3.25 |
| ParaHippocampal Gyrus | R | 22 | 10 | -22 |  |  | 3.08 | 3.04 |
| Rolandic Operculum | L | -56 | -2 | 8 | 0.93 | 87 | 3.44 | 3.38 |
| Insula Lobe | R | 34 | 8 | 12 | 0.93 | 52 | 3.44 | 3.37 |
| Insula Lobe | L | -32 | 2 | 16 | 0.93 | 28 | 3.39 | 3.33 |
| Middle Orbital Gyrus | L | -24 | 40 | -16 | 0.93 | 38 | 3.33 | 3.28 |
|  |  | 28 | 40 | -10 | 0.93 | 20 | 3.33 | 3.27 |
| Superior Parietal Lobule | L | -18 | -48 | 70 | 0.93 | 82 | 3.32 | 3.26 |
| Postcentral Gyrus | L | -28 | -44 | 68 |  |  | 2.86 | 2.82 |
|  |  | 2 | 14 | 20 | 0.93 | 16 | 3.04 | 2.99 |
| Inferior Parietal Lobule | R | 52 | -38 | 52 | 0.93 | 10 | 2.99 | 2.95 |
| Middle Frontal Gyrus | R | 40 | 22 | 46 | 0.93 | 33 | 2.95 | 2.91 |
| Superior Frontal Gyrus | R | 30 | 28 | 54 |  |  | 2.71 | 2.68 |
| Fusiform Gyrus | L | -42 | -66 | -16 | 0.93 | 11 | 2.93 | 2.89 |
| Middle Occipital Gyrus | L | -50 | -76 | 0 | 0.93 | 28 | 2.92 | 2.88 |
| Thalamus | R | 14 | -14 | 6 | 0.93 | 12 | 2.89 | 2.85 |

Brain regions in young adults showing increased activation in response to painful compared to warm stimuli at *p*(unc) = .005 and *k* ≥ 10 and cluster correction FDR p-levels indicated separately.
