# Supplementary material for: Better Executive Functions Are Associated With More Efficient Cognitive Pain Modulation in Older Adults: An fMRI Study
Source: Front Aging Neurosci. 2022 Jul 7;14:828742. doi: 10.3389/fnagi.2022.828742 (PMC9302198; doi:10.3389/fnagi.2022.828742)
Supplement: Supplementary file 7 [file Table_7.DOCX]

**Table S7: Neural pain response in OA.**

| Anatomical label |  | MNI coordinates | | | Cluster | | | |
| --- | --- | --- | --- | --- | --- | --- | --- | --- |
|  |  | x | y | z | *p*(FDR-corr) | *k* | *T* | *Z* |
| SupraMarginal Gyrus | R | 50 | -24 | 24 | 0.00 | 2230 | 6.11 | 5.80 |
| Rolandic Operculum | R | 58 | 6 | 6 |  |  | 5.64 | 5.40 |
| Insula Lobe | R | 36 | -16 | 18 |  |  | 5.04 | 4.86 |
| Postcentral Gyrus | R | 22 | -42 | 72 | 0.00 | 1766 | 5.97 | 5.69 |
| Superior Frontal Gyrus | R | 20 | -14 | 72 |  |  | 5.01 | 4.84 |
| Precentral Gyrus | R | 32 | -26 | 70 |  |  | 4.84 | 4.68 |
| Superior Orbital Gyrus | R | 24 | 34 | -14 | 0.62 | 212 | 5.30 | 5.10 |
| IFG p. Orbitalis | R | 36 | 26 | -14 |  |  | 2.69 | 2.66 |
| Superior Temporal Gyrus | L | -64 | -28 | 22 | 0.31 | 397 | 4.88 | 4.72 |
| IFG p. Orbitalis | L | -22 | 34 | -12 | 0.41 | 286 | 4.46 | 4.33 |
| Insula Lobe | L | -24 | 20 | -14 |  |  | 3.82 | 3.74 |
| IFG p. Orbitalis | L | -30 | 24 | -10 |  |  | 2.90 | 2.86 |
| MCC | L | -4 | 16 | 34 | 0.33 | 349 | 3.81 | 3.73 |
| MCC | R | 6 | 8 | 40 |  |  | 2.77 | 2.74 |
| Rectal Gyrus | R | 20 | 12 | -16 | 0.93 | 58 | 3.79 | 3.71 |
| Rolandic Operculum | L | -56 | 0 | 8 | 0.93 | 123 | 3.71 | 3.63 |
| Superior Parietal Lobule | L | -18 | -44 | 72 | 0.93 | 78 | 3.62 | 3.55 |
| Lingual Gyrus | L | -30 | -50 | -4 | 0.93 | 129 | 3.58 | 3.51 |
| Fusiform Gyrus | L | -34 | -52 | -12 |  |  | 3.34 | 3.29 |
| Area hOc1 [V1] |  | -18 | -78 | 0 | 0.93 | 99 | 3.41 | 3.35 |
| Calcarine Gyrus | L | -10 | -84 | 2 |  |  | 3.07 | 3.03 |
| Insula Lobe | L | -34 | 6 | 10 | 0.93 | 24 | 3.38 | 3.32 |
| Inferior Parietal Lobule | L | -56 | -46 | 44 | 0.93 | 36 | 3.27 | 3.22 |
| Inferior Parietal Lobule | L | -50 | -54 | 48 |  |  | 2.82 | 2.79 |
| Middle Temporal Gyrus | L | -44 | -54 | 8 | 0.93 | 105 | 3.25 | 3.20 |
| Posterior-Medial Frontal | R | 6 | -18 | 50 | 0.93 | 86 | 3.21 | 3.16 |
| Posterior-Medial Frontal | R | 6 | -10 | 54 |  |  | 2.95 | 2.91 |
|  |  | 38 | 0 | -4 | 0.93 | 28 | 3.18 | 3.13 |
| Precentral Gyrus | R | 44 | 4 | 50 | 0.93 | 70 | 3.13 | 3.08 |
| Posterior-Medial Frontal | R | 6 | 14 | 68 | 0.93 | 21 | 3.07 | 3.03 |
| IFG p. Triangularis | R | 34 | 26 | 10 | 0.93 | 54 | 3.06 | 3.02 |
|  |  | 28 | 22 | 14 |  |  | 2.71 | 2.68 |
| Middle Temporal Gyrus | R | 60 | -56 | 6 | 0.93 | 13 | 3.05 | 3.01 |
| Paracentral Lobule | L | -16 | -32 | 68 | 0.93 | 23 | 3.05 | 3.00 |
| Insula Lobe | R | 36 | 6 | 12 | 0.93 | 14 | 2.91 | 2.88 |
| Middle Temporal Gyrus | L | -66 | -38 | 0 | 0.93 | 18 | 2.88 | 2.84 |
|  |  | 0 | -4 | -16 | 0.93 | 12 | 2.87 | 2.83 |
| Superior Medial Gyrus | R | 8 | 38 | 54 | 0.93 | 11 | 2.76 | 2.72 |

Brain regions in older adults showing increased activation in response to painful compared to warm stimuli at *p*(unc) = .005 and *k* ≥ 10 and cluster correction FDR p-levels indicated separately.
