# Supplementary material for: Better Executive Functions Are Associated With More Efficient Cognitive Pain Modulation in Older Adults: An fMRI Study
Source: Front Aging Neurosci. 2022 Jul 7;14:828742. doi: 10.3389/fnagi.2022.828742 (PMC9302198; doi:10.3389/fnagi.2022.828742)
Supplement: Supplementary file 8 [file Table_8.DOCX]

**Table S8: Neural distraction effect in young adults.**

| Anatomical labels |  | MNI coordinates | | | Cluster | | | |
| --- | --- | --- | --- | --- | --- | --- | --- | --- |
|  |  | x | y | z | *p*(FDR-corr) | *k* | *T* | *Z* |
| Superior Parietal Lobule | R | 38 | -52 | 58 | 0.00 | 8472 | 5.31 | 5.10 |
|  |  | 30 | 6 | 40 |  |  | 4.34 | 4.22 |
|  |  | -28 | -38 | 42 |  |  | 4.21 | 4.10 |
| Middle Temporal Gyrus | R | 52 | -36 | -8 | 0.10 | 684 | 4.41 | 4.29 |
| Middle Temporal Gyrus | R | 54 | -24 | -12 |  |  | 3.86 | 3.77 |
| Middle Temporal Gyrus | R | 62 | -22 | -12 |  |  | 3.36 | 3.30 |
| Caudate Nucleus | R | 12 | -2 | 20 | 0.56 | 299 | 3.83 | 3.74 |
| Caudate Nucleus | R | 18 | 10 | 16 |  |  | 3.47 | 3.41 |
|  |  | 18 | 6 | 26 |  |  | 3.16 | 3.11 |
| Middle Frontal Gyrus | R | 38 | 38 | 36 | 0.93 | 140 | 3.75 | 3.68 |
| Middle Frontal Gyrus | R | 32 | 38 | 42 |  |  | 3.19 | 3.14 |
| Inferior Temporal Gyrus | L | -42 | -30 | -22 | 0.93 | 186 | 3.75 | 3.67 |
| Inferior Temporal Gyrus | L | -52 | -18 | -28 |  |  | 3.73 | 3.66 |
| Inferior Temporal Gyrus | L | -52 | -26 | -22 |  |  | 3.21 | 3.16 |
| Superior Orbital Gyrus | R | 18 | 38 | -18 | 0.93 | 91 | 3.67 | 3.60 |
| Superior Orbital Gyrus | R | 24 | 42 | -14 |  |  | 3.37 | 3.31 |
| Postcentral Gyrus | R | 64 | -12 | 30 | 0.56 | 307 | 3.36 | 3.30 |
| SupraMarginal Gyrus | R | 62 | -30 | 42 |  |  | 3.20 | 3.15 |
| Rolandic Operculum | R | 54 | -12 | 16 |  |  | 3.11 | 3.06 |
| Superior Temporal Gyrus | R | 68 | -16 | 0 | 0.93 | 38 | 3.34 | 3.28 |
| Superior Temporal Gyrus | R | 64 | -8 | 2 |  |  | 2.77 | 2.74 |
| Middle Temporal Gyrus | R | 68 | -28 | -2 |  |  | 2.70 | 2.67 |
| IFG p. Opercularis | R | 60 | 16 | 26 | 0.93 | 31 | 3.34 | 3.28 |
|  |  | 40 | -42 | 26 | 0.93 | 63 | 3.21 | 3.16 |
| IFG p. Orbitalis | R | 32 | 26 | -20 | 0.93 | 29 | 3.20 | 3.15 |
| IFG p. Orbitalis | R | 40 | 30 | -18 |  |  | 2.99 | 2.95 |
| IFG p. Orbitalis | L | -46 | 34 | -12 | 0.93 | 51 | 3.17 | 3.12 |
| Superior Orbital Gyrus | R | 24 | 58 | -4 | 0.93 | 11 | 3.15 | 3.10 |
|  |  | -4 | -36 | 14 | 0.93 | 18 | 3.03 | 2.99 |
| Angular Gyrus | R | 60 | -52 | 28 | 0.93 | 26 | 3.01 | 2.97 |
| SupraMarginal Gyrus | R | 62 | -42 | 32 |  |  | 2.75 | 2.72 |
| Caudate Nucleus | L | -14 | 26 | 0 | 0.93 | 14 | 3.00 | 2.96 |
| Middle Temporal Gyrus | R | 64 | -10 | -24 | 0.93 | 10 | 3.00 | 2.96 |
| Middle Frontal Gyrus | L | -44 | 4 | 52 | 0.93 | 11 | 2.94 | 2.90 |
| Inferior Temporal Gyrus | R | 52 | -68 | -8 | 0.93 | 13 | 2.93 | 2.89 |
| Insula Lobe | L | -30 | 16 | 4 | 0.93 | 15 | 2.90 | 2.86 |

Brain regions in young adults showing reduced activation in response to painful stimuli during the high load task when compared to the low load task (contrast: *(pain > warm) _low load_ > (pain > warm) _high load_* ) at *p*(unc) = .005 and *k* ≥ 10 and cluster correction FDR p-levels indicated separately.
