# Supplementary material for: Better Executive Functions Are Associated With More Efficient Cognitive Pain Modulation in Older Adults: An fMRI Study
Source: Front Aging Neurosci. 2022 Jul 7;14:828742. doi: 10.3389/fnagi.2022.828742 (PMC9302198; doi:10.3389/fnagi.2022.828742)
Supplement: Supplementary file 9 [file Table_9.DOCX]

| Anatomical labels |  | MNI coordinates | | | Cluster | | | |
| --- | --- | --- | --- | --- | --- | --- | --- | --- |
|  |  | x | y | z | *p*(FDR-corr) | *K* | *T* | *Z* |
| Fusiform Gyrus | L | -44 | -56 | -18 | 0.93 | 189 | 3.96 | 3.87 |
| Fusiform Gyrus | L | -40 | -66 | -16 |  |  | 3.47 | 3.41 |
|  |  | -38 | -58 | -8 |  |  | 3.18 | 3.13 |
|  |  | 32 | -76 | 10 | 0.93 | 344 | 3.44 | 3.38 |
| Calcarine Gyrus | R | 16 | -88 | 8 |  |  | 3.26 | 3.21 |
| Inferior Temporal Gyrus | R | 52 | -64 | -10 |  |  | 3.22 | 3.17 |
| Insula Lobe | R | 28 | 12 | -20 | 0.93 | 142 | 3.42 | 3.36 |
| Precuneus | R | 18 | -62 | 42 | 0.93 | 218 | 3.42 | 3.36 |
| Cuneus | R | 24 | -68 | 26 |  |  | 3.11 | 3.06 |
| Superior Parietal Lobule | R | 26 | -70 | 50 |  |  | 2.82 | 2.79 |
| Superior Parietal Lobule | L | -12 | -74 | 40 | 0.93 | 83 | 3.20 | 3.15 |
| Inferior Occipital Gyrus | L | -28 | -82 | -6 | 0.93 | 22 | 3.17 | 3.12 |
| Middle Occipital Gyrus | L | -34 | -86 | -2 |  |  | 2.83 | 2.80 |
|  |  | -14 | -22 | -14 | 0.93 | 44 | 3.12 | 3.07 |
|  |  | -40 | -4 | -26 | 0.93 | 14 | 2.97 | 2.93 |
|  |  | -28 | 2 | -14 | 0.93 | 17 | 2.95 | 2.91 |
| Olfactory cortex | L | -12 | 10 | -16 | 0.93 | 18 | 2.94 | 2.90 |
| Middle Temporal Gyrus | L | -54 | -68 | 4 | 0.93 | 14 | 2.91 | 2.87 |
|  |  | -32 | -18 | -8 | 0.93 | 10 | 2.88 | 2.84 |
| Middle Occipital Gyrus | L | -34 | -68 | 24 | 0.93 | 10 | 2.82 | 2.79 |
| Calcarine Gyrus | L | -16 | -80 | 6 | 0.93 | 15 | 2.82 | 2.78 |

**Table S9: Neural distraction effect in older adults.**

Brain regions in older adults showing reduced activation in response to painful stimuli during the high load task when compared to the low load task (contrast: *(pain > warm) _low load_ > (pain > warm) _high load_*) at *p*(unc) = .005 and *k* ≥ 10 and cluster correction FDR p-levels indicated separately.
