# Supplementary material for: Better Executive Functions Are Associated With More Efficient Cognitive Pain Modulation in Older Adults: An fMRI Study
Source: Front Aging Neurosci. 2022 Jul 7;14:828742. doi: 10.3389/fnagi.2022.828742 (PMC9302198; doi:10.3389/fnagi.2022.828742)
Supplement: Supplementary file 10 [file Table_10.DOCX]

**Table S10. Correlations between the neural and behavioral distraction effect.**

|  |  | MNI coordinates | | | Cluster | | | |
| --- | --- | --- | --- | --- | --- | --- | --- | --- |
| Anatomical labels |  | x | y | z | p(FDR-corr) | *k* | *T* | *Z* |
| Young adults (DE-I) ^a^ |  |  |  |  |  |  |  |  |
| Inferior temporal gyrus | L | -58 | -50 | -12 | 0.75 | 44 | 4.44 | 4.09 |
| Inferior temporal gyrus | L | -52 | -56 | -18 |  |  | 3.42 | 3.24 |
| Angular gyrus | L | -44 | -72 | 38 | 0.75 | 61 | 4.25 | 3.94 |
| Precentral gyrus | R | 30 | -16 | 70 | 0.75 | 41 | 4.16 | 3.87 |
| Mid orbital gyrus | L | -10 | 46 | -12 | 0.75 | 66 | 3.99 | 3.73 |
| Rectal gyrus | R | 4 | 50 | -18 |  |  | 3.89 | 3.65 |
| Young adults (DE-U) ^b^ |  | - | - | - | - | - | - | - |
| Older adults (DE-I) ^a^ |  | - | - | - | - | - | - | - |
| Older adults (DE-U) ^b^ |  |  |  |  |  |  |  |  |
| Postcentral gyrus | L | -62 | -12 | 38 | 0.88 | 75 | 4.42 | 4.08 |
| Middle frontal gyrus | L | -36 | 26 | 48 | 0.88 | 28 | 3.99 | 3.72 |
| Middle frontal gyrus | R | 36 | 10 | 60 | 0.88 | 22 | 3.85 | 3.61 |
| Paracentral lobule | R | 4 | -30 | 78 | 0.88 | 23 | 3.68 | 3.46 |
| Postcentral gyrus | R | 12 | -34 | 74 |  |  | 3.34 | 3.17 |

^a^ DE-I: Behavioral distraction effect on the intensity scale; ^b^ Behavioral distraction effect on the unpleasantness scale; Regions showing a positive correlation between the neural distraction effect size and the behavioral distraction effect size (DE-I and DE-U) in YA and OA at *p*(unc) = .001 and *k* ≥ 20 (t-test model), and cluster correction FDR *p*-levels indicated separately.
