# Supplementary material for: Better Executive Functions Are Associated With More Efficient Cognitive Pain Modulation in Older Adults: An fMRI Study
Source: Front Aging Neurosci. 2022 Jul 7;14:828742. doi: 10.3389/fnagi.2022.828742 (PMC9302198; doi:10.3389/fnagi.2022.828742)
Supplement: Supplementary file 11 [file Table_11.DOCX]

| Anatomical labels |  | MNI coordinates | | |  | Cluster | | |
| --- | --- | --- | --- | --- | --- | --- | --- | --- |
|  |  | x | y | z |  | *k* | *T* | *Z* |
| Rectal gyrus | R | 8 | 41 | -26 |  | 139860 | 12.77 | 65535.00 |
| Superior temporal gyrus | L | -47 | -12 | -3 |  |  | 11.65 | 65535.00 |
| Middle frontal gyrus | R | 41 | 11 | 38 |  |  | 11.02 | 65535.00 |
| Cerebelum X | R | 24 | -42 | -44 |  | 43 | 5.72 | 5.06 |
| Middle occipital gyrus | R | 36 | -68 | 38 |  | 61 | 5.70 | 5.05 |
| Cuneus | R | 18 | -95 | 14 |  | 42 | 5.54 | 4.94 |
| Angular gyrus | L | -48 | -60 | 33 |  | 156 | 5.54 | 4.93 |
| Middle occipital gyrus | L | -24 | -92 | 5 |  | 95 | 5.46 | 4.87 |
|  |  |  |  |  |  |  |  |  |
| Middle occipital gyrus | L | -38 | -87 | 8 |  |  | 5.22 | 4.70 |
| Superior parietal lobule | L | -23 | -44 | 65 |  | 64 | 5.43 | 4.85 |
| Cerebelum IX | R | 15 | -47 | -45 |  | 45 | 5.37 | 4.81 |
| Cerebelum IX | R | 15 | -57 | -51 |  |  | 4.99 | 4.52 |

**Table S11: Age-related differences (YA > OA) in GM volume.**

YA = young adults; OA = older adults; Grey matter (GM) volume was corrected for the total intracranial volume for each participant. FWE-corrected contrast at *p* = .05 and *k* ≥ 20.
