# Supplementary material for: Better Executive Functions Are Associated With More Efficient Cognitive Pain Modulation in Older Adults: An fMRI Study
Source: Front Aging Neurosci. 2022 Jul 7;14:828742. doi: 10.3389/fnagi.2022.828742 (PMC9302198; doi:10.3389/fnagi.2022.828742)
Supplement: Supplementary file 12 [file Table_12.DOCX]

**Table S12: Neural distraction effect for young adults > older adults.**

|  | Distraction effect (YA > OA) with no covariate | | | | | | | | | |  |  | Distraction effect (YA > OA) with total GM volume as covariate | | | | | | | | |
| --- | --- | --- | --- | --- | --- | --- | --- | --- | --- | --- | --- | --- | --- | --- | --- | --- | --- | --- | --- | --- | --- |
| Anatomical labels | | |  | MNI coordinates | | | Cluster | | | |  | Anatomical labels | |  | MNI coordinates | | | Cluster | | | |
|  | | |  | x | y | z | *p*(FDR-corr) | *K* | *T* | *Z* |  |  | |  | x | y | z | *p*(FDR-corr) | *k* | *T* | *Z* |
| Superior Medial Gyrus | | R | | 6 | 36 | 42 | 0.92 | 197 | 3.77 | 3.55 |  | Superior Medial Gyrus | | R | 6 | 36 | 42 | 0.92 | 227 | 3.73 | 3.51 |
| Superior Medial Gyrus | | R | | 6 | 38 | 52 |  |  | 3.06 | 2.93 |  | Superior Medial Gyrus | | R | 6 | 38 | 52 |  |  | 3.24 | 3.09 |
| Superior Medial Gyrus | | R | | 2 | 28 | 46 |  |  | 2.88 | 2.78 |  | Superior Medial Gyrus | | R | 2 | 28 | 46 |  |  | 2.92 | 2.81 |
| Postcentral Gyrus | | L | | -50 | -18 | 32 | 0.92 | 84 | 3.18 | 3.04 |  | MCC | | R | 12 | -20 | 44 | 0.92 | 20 | 3.18 | 3.04 |
| SupraMarginal Gyrus | | L | | -54 | -24 | 36 |  |  | 3.10 | 2.97 |  | Postcentral Gyrus | | L | -50 | -18 | 32 | 0.92 | 76 | 3.15 | 3.01 |
| Postcentral Gyrus | | L | | -44 | -24 | 40 |  |  | 2.89 | 2.79 |  | SupraMarginal Gyrus | | L | -54 | -24 | 36 |  |  | 3.07 | 2.94 |
|  | |  | | -14 | -4 | 36 | 0.92 | 11 | 3.17 | 3.03 |  | Postcentral Gyrus | | L | -44 | -24 | 40 |  |  | 2.85 | 2.74 |
| MCC | | R | | 12 | -20 | 44 | 0.92 | 19 | 3.14 | 3.00 |  |  | |  | 6 | -6 | 20 | 0.92 | 36 | 3.13 | 2.99 |
| SupraMarginal Gyrus | | R | | 56 | -34 | 26 | 0.92 | 12 | 3.12 | 2.99 |  | Caudate Nucleus | | R | 14 | 0 | 22 |  |  | 2.74 | 2.64 |
|  | |  | | 6 | -6 | 20 | 0.92 | 40 | 3.12 | 2.98 |  |  | |  | -14 | -4 | 36 | 0.92 | 10 | 3.12 | 2.98 |
| Caudate Nucleus | | R | | 14 | 0 | 22 |  |  | 2.75 | 2.66 |  | SupraMarginal Gyrus | | R | 56 | -34 | 26 | 0.92 | 11 | 3.11 | 2.97 |
| Heschls Gyrus | | R | | 64 | -4 | 6 | 0.92 | 23 | 3.11 | 2.98 |  | Heschls Gyrus | | R | 64 | -4 | 6 | 0.92 | 22 | 3.10 | 2.97 |
| SupraMarginal Gyrus | | L | | -62 | -32 | 24 | 0.92 | 22 | 3.11 | 2.98 |  | SupraMarginal Gyrus | | L | -62 | -32 | 24 | 0.92 | 18 | 3.10 | 2.96 |
| ACC | | L | | -4 | 36 | 0 | 0.92 | 19 | 3.01 | 2.89 |  | Rolandic Operculum | | R | 48 | -16 | 14 | 0.92 | 36 | 3.01 | 2.88 |
| Rolandic Operculum | | R | | 46 | -16 | 14 | 0.92 | 36 | 3.01 | 2.89 |  | Rolandic Operculum | | R | 44 | -8 | 18 |  |  | 2.78 | 2.68 |
| Rolandic Operculum | | R | | 44 | -8 | 18 |  |  | 2.77 | 2.68 |  | ACC | | L | -4 | 36 | 0 | 0.92 | 17 | 2.98 | 2.86 |

YA: young adults; OA: older adults. Brain regions in young adults > older adults showing reduced activation in response to painful stimuli during the high load task when compared to the low load task (contrast: *(pain > warm) _low load_ > (pain > warm) _high load_* ) at *p*(unc) = .005 and *k* ≥ 10 and cluster correction FDR p-levels indicated separately. Note that the model is based on a t-test model (to allow for the inclusion of covariates) and not a flexible factorial model (as used in the main manuscript). The results to the left show the results of a model with no covariate (which may thus differ slightly from the results reported in Table 2 in the manuscript); the results to the right show the results of a model with total grey matter (GM) volume (corrected for total intracranial volume) as covariate.
