# Supplementary material for: Better Executive Functions Are Associated With More Efficient Cognitive Pain Modulation in Older Adults: An fMRI Study
Source: Front Aging Neurosci. 2022 Jul 7;14:828742. doi: 10.3389/fnagi.2022.828742 (PMC9302198; doi:10.3389/fnagi.2022.828742)
Supplement: Supplementary file 14 [file Table_14.DOCX]

**Table S14:** **Neural distraction effect based on ROI analyses.**

|  | Anatomical regions |  | MNI coordinates | | | Cluster | | | |  |
| --- | --- | --- | --- | --- | --- | --- | --- | --- | --- | --- |
|  |  |  |  |  |  |  |  |  |  |  |
|  |  |  | x | y | z | *p*(FDR-corr) | *K* | *T* | *Z* |  |
| *Across groups* |  |  |  |  |  |  |  |  |  |  |
|  | Insula Lobe | R | 30 | 24 | -20 | 0.93 | 37 | 3.9 | 3.81 |  |
|  | Insula Lobe | R | 26 | 14 | -18 |  |  | 3.22 | 3.17 |  |
|  | Postcentral Gyrus | R | 40 | -44 | 62 | 0.6 | 199 | 3.77 | 3.69 |  |
|  | Postcentral Gyrus | R | 32 | -30 | 60 |  |  | 3.57 | 3.5 |  |
|  | MCC | R | 10 | 12 | 44 | 0.6 | 171 | 3.58 | 3.51 |  |
|  | MCC | L | -10 | 6 | 42 |  |  | 3.33 | 3.27 |  |
|  | MCC | L | -8 | 2 | 32 |  |  | 2.98 | 2.94 |  |
|  | Postcentral Gyrus | L | -46 | -40 | 58 | 0.93 | 63 | 3.26 | 3.2 |  |
|  | Postcentral Gyrus | L | -30 | -40 | 44 |  |  | 3.22 | 3.17 |  |
|  | Postcentral Gyrus | L | -32 | -40 | 52 |  |  | 2.84 | 2.8 |  |
|  | Postcentral Gyrus | R | 64 | -18 | 32 | 0.93 | 20 | 3.21 | 3.16 |  |
|  | Postcentral Gyrus | R | 14 | -52 | 72 | 0.93 | 13 | 3.1 | 3.05 |  |
|  | Postcentral Gyrus | R | 58 | -24 | 50 | 0.93 | 15 | 3.02 | 2.97 |  |
|  | MCC | R | 6 | -30 | 36 | 0.93 | 30 | 2.92 | 2.88 |  |
|  | Insula Lobe | L | -38 | 20 | 4 | 0.93 | 15 | 2.87 | 2.83 |  |
|  | Postcentral Gyrus | R | 24 | -38 | 74 | 0.93 | 20 | 2.85 | 2.82 |  |
| *YA* |  |  |  |  |  |  |  |  |  |  |
|  | Postcentral Gyrus | R | 40 | -44 | 62 | 0.93 | 57 | 4.02 | 3.93 |  |
|  | Postcentral Gyrus | L | -30 | -38 | 44 | 0.93 | 192 | 3.93 | 3.84 |  |
|  | Postcentral Gyrus | L | -32 | -36 | 52 |  |  | 3.65 | 3.58 |  |
|  | Postcentral Gyrus | L | -44 | -40 | 56 |  |  | 3.02 | 2.98 |  |
|  | MCC | L | -10 | 10 | 44 | 0.1 | 938 | 3.63 | 3.55 |  |
|  | MCC | R | 4 | 26 | 40 |  |  | 3.62 | 3.55 |  |
|  | MCC | L | -2 | 6 | 42 |  |  | 3.57 | 3.51 |  |
|  | Insula Lobe | R | 44 | 4 | 8 | 0.93 | 202 | 3.56 | 3.5 |  |
|  | Insula Lobe | R | 40 | 16 | 2 |  |  | 3.18 | 3.13 |  |
|  | Insula Lobe | R | 42 | 24 | 0 |  |  | 3.01 | 2.97 |  |
|  | MCC | L | -14 | -36 | 54 | 0.93 | 42 | 3.51 | 3.44 |  |
|  | Postcentral Gyrus | R | 28 | -32 | 60 | 0.93 | 169 | 3.5 | 3.44 |  |
|  | Postcentral Gyrus | R | 12 | -34 | 60 |  |  | 3.35 | 3.29 |  |
|  | Postcentral Gyrus | R | 34 | -36 | 48 |  |  | 2.82 | 2.78 |  |
|  | Postcentral Gyrus | L | -62 | -20 | 36 | 0.93 | 112 | 3.43 | 3.37 |  |
|  | Postcentral Gyrus | L | -54 | -24 | 30 |  |  | 3.13 | 3.08 |  |
|  | Postcentral Gyrus | L | -64 | -20 | 26 |  |  | 3 | 2.96 |  |
|  | Postcentral Gyrus | R | 64 | -12 | 30 | 0.93 | 103 | 3.36 | 3.3 |  |
|  | Postcentral Gyrus | R | 54 | -22 | 42 |  |  | 2.91 | 2.88 |  |
|  | Postcentral Gyrus | R | 56 | -10 | 20 |  |  | 2.9 | 2.86 |  |
|  | Insula Lobe | L | -30 | 16 | 4 | 0.93 | 12 | 2.9 | 2.86 |  |
| *OA* |  |  |  |  |  |  |  |  |  |  |
|  | Insula Lobe | R | 28 | 12 | -20 | 0.93 | 49 | 3.42 | 3.36 |  |
| *YA > OA* |  |  |  |  |  |  |  |  |  |  |
|  | ACC | L | -4 | 36 | 0 | 0.93 | 28 | 3.28 | 3.23 |  |
|  | Insula Lobe | R | 42 | -12 | 14 | 0.93 | 44 | 3.15 | 3.1 |  |
|  | Insula Lobe | R | 44 | 8 | 4 | 0.93 | 111 | 3.13 | 3.09 |  |
|  | Insula Lobe | R | 44 | 0 | 8 |  |  | 2.93 | 2.89 |  |
|  | Insula Lobe | R | 38 | 16 | -2 |  |  | 2.91 | 2.87 |  |
|  | Postcentral Gyrus | L | -48 | -20 | 32 | 0.93 | 35 | 3.06 | 3.01 |  |
|  | Postcentral Gyrus | L | -44 | -24 | 38 |  |  | 2.92 | 2.88 |  |
|  | MCC | R | 12 | -14 | 42 | 0.93 | 15 | 3.02 | 2.98 |  |
| *OA > YA* | - | - | - | - | - | - | - | - | - |  |

Brain regions showing reduced activation in response to painful stimuli during the high load task when compared to the low load task (contrast: *(pain > warm) _low load_ > (pain > warm) _high load_*) at *p*(unc) = .005 and *k* ≥ 10 and cluster correction FDR p-levels indicated separately (note that p-values were adjusted for search volume). Brain regions were based on anatomical masks from the aal atlas (i.e., the bilateral insula, bilateral thalamus, bilateral postcentral gyrus and anterior and mid cingulate cortex).
