# Supplementary material for: Better Executive Functions Are Associated With More Efficient Cognitive Pain Modulation in Older Adults: An fMRI Study
Source: Front Aging Neurosci. 2022 Jul 7;14:828742. doi: 10.3389/fnagi.2022.828742 (PMC9302198; doi:10.3389/fnagi.2022.828742)
Supplement: Supplementary file 15 [file Table_15.DOCX]

|  | Anatomical regions |  | | MNI coordinates | | | Cluster | | | |  |
| --- | --- | --- | --- | --- | --- | --- | --- | --- | --- | --- | --- |
|  |  |  | | x | y | z | *p*(FDR-corr) | *K* | *T* | *Z* | |
| *Across groups* | - |  | | - | - | - | - | - | - | - | |
| *YA* |  |  | |  |  |  |  |  |  |  | |
|  | Superior Medial Gyrus | | L | -10 | 54 | 22 | 0.46 | 34 | 3.00 | 2.96 | |
|  | Superior Medial Gyrus | | L | -12 | 50 | 12 |  |  | 2.84 | 2.80 | |
| *OA* |  | |  |  |  |  |  |  |  |  | |
|  | ACC | | L | -4 | 36 | 2 | 0.80 | 108 | 3.50 | 3.44 | |
|  | ACC | | R | 4 | 38 | 0 |  |  | 3.19 | 3.14 | |
| *YA > OA* | - | |  | - | - | - | - | - | - | - | |
| *OA > YA* | Superior Medial Gyrus | | R | 4 | 38 | 44 | 0.89 | 191 | 3.39 | 3.33 | |
|  | Superior Medial Gyrus | | R | 6 | 38 | 52 |  |  | 2.96 | 2.92 | |
|  | Superior Medial Gyrus | | R | 6 | 26 | 50 |  |  | 2.67 | 2.64 | |
|  | ACC | | L | -4 | 36 | 0 | 0.93 | 28 | 3.28 | 3.23 | |
|  | MCC | | R | 12 | -14 | 42 | 0.93 | 15 | 3.02 | 2.98 | |
|  | IFG p. Opercularis | | R | 42 | 8 | 8 | 0.93 | 11 | 2.97 | 2.93 | |
|  | Superior Frontal Gyrus | | R | 20 | 56 | 4 | 0.93 | 19 | 2.88 | 2.85 | |

**Table 15: Neural distraction mechanism based on ROIs.**

Brain regions showing increased activation in response to painful stimuli during the high load task when compared to the low load task (contrast: *(pain > warm) _high load_ > (pain > warm) _low load_*) at *p*(unc) = .005 and *k* ≥ 10 and cluster correction FDR p-levels indicated separately (note that p-values were adjusted for search volume). Brain regions were based on anatomical masks from the aal atlas (i.e., the prefrontal cortex and anterior and mid cingulate cortex).
