# Supplementary material for: Better Executive Functions Are Associated With More Efficient Cognitive Pain Modulation in Older Adults: An fMRI Study
Source: Front Aging Neurosci. 2022 Jul 7;14:828742. doi: 10.3389/fnagi.2022.828742 (PMC9302198; doi:10.3389/fnagi.2022.828742)
Supplement: Supplementary file 16 [file Data_Sheet_1.docx]

Supplementary Material

# Methods

## Calibration procedure for n-back task speed

The task speed was calibrated in a procedure adapted from Buhle and Wager (2010) and previously described in Rischer et al. (2020). During this calibration, the time between presentation of consecutive letters was adjusted every two trials (one trial consisting of 20 seconds of the task) based on the participant’s performance. There were 20 trials, and thus 10 adaptive steps during which the interval was either increased or decreased. We used the non-parametric measure, A’, as an index of the participants’ performance, with an A′ value of 1 indicating perfect performance, and an A′ value of 0.5 meaning performance at chance (Stanislaw and Todorov, 1999). Our target level of performance was set to an A’ value of 0.85 to maintain a similar level of task difficulty across participants. If A′ was greater than 0.85 and the missing response rate was <25%, the inter-character interval duration was reduced by the absolute value of 1.200*(A′−0.85). In case A′ was equal to or smaller than 0.85, the duration was increased by the absolute value of 600*(A′−0.85). The inter-character interval was also increased if the missing response rate was equal or greater than 25%, even if A’ was equal or greater than 0.85 as missing responses had no effect on the false alarm rate and did thus not affect the A’ values. In this case, the interval duration was increased by an absolute value of 600*(A’-1.00). A constriction prevented the interval duration from decreasing below 100 ms. We also considered responses with an RT < 150 ms as incorrect as they are unlikely to reflect true responses (Schmiedek et al., 2009; Legrain et al., 2011). In addition, the first two letters of the high load task were excluded from the analyses, as no comparison could be made with letters presented two steps back. Note that the initial presentation speed was set to one letter every 2,000 ms but could be increased by the experimenter if participants showed difficulties to respond in time during the practice phase (eight OA started with an adapted speed of 2,500 ms per letter and one OA with a speed of 3,000 ms per letter).

## Calibration procedure for thermal stimuli

Innocuous warm and moderately painful stimuli were selected based on a calibration procedure outside the scanner. The thermal stimulator was attached to the participants’ left forearm and participants were presented with three practice stimuli of 40°C, 45°C and 46.5°C to familiarize them with the range and duration of the stimuli and the rating procedure. Participants were asked to rate the perceived intensity and unpleasantness of the stimuli on 200-point scales. The intensity scale ranged from “No warmth” (0) over “Just pain” (100) to “Unbearable pain” (200) and the unpleasantness scale ranged from “Very pleasant” (0) to “Very unpleasant” (200). Our target ratings for the warm and painful stimuli were 60 and 140 respectively.

During the calibration, YA were presented with 14 stimuli in pseudorandomized order, ranging between 42°C and 48°C (in increments of 0.5°C). Two more stimuli (48.5°C and 49°C) could be administered if the target rating of 140 points on the 200-point intensity scale was not reached during the calibration. As previous research indicated that OA may differ from YA in terms of pain sensitivity (Lautenbacher et al., 2017), OA were initially only presented with 12 stimuli, ranging between 42°C and 47°C. The experimenter could administer four more stimuli (47.5°C to 49°C in increments of 0.5°C) if the target intensity rating was not reached.

The experimenter determined the target temperature for warm and painful stimuli by interpolating the resulting intensity ratings with the TREND function in MS Excel 2016 (Microsoft Excel) and by selecting the temperatures corresponding to an intensity rating of 60 points and 140 points for the warm and painful stimuli, respectively.

## Distraction paradigm settings

In both the low and high load condition, ca. 25% of all letters (either C, F, J, N, Q, S, V or X) were targets. In addition, the high load task contained ca. 12.5% lures (i.e., letters that were identical to the one presented one or three steps back) to increase task difficulty. In both tasks, no more than two target letters were shown consecutively. Each letter was presented for 500 ms, preceded by a fixation cross (250 ms) and followed by a blank inter-character interval (adaptive duration). All letters in the low load task were presented in yellow and all letters in the high load task in magenta (both on a black background) in order to help participants to distinguish between both tasks (after the cue word had been presented). As in the calibration procedure, the duration of the inter-character interval in the high load task was continuously adapted every two trials (starting with the duration derived after the calibration). The duration of the inter-character interval in the low load task was set to the same duration as the high load task in between blocks.

## Post-experimental questionnaire

Participants rated different aspects of the experiment on 11-point Likert scales, ranging from “not at all” (0) to “extremely” or “a lot” (10). A list of all questions can be found below (where the “X-target task” refers to the low-load 0-back task).

1. How much attention did you pay to the thermal stimuli?
2. How difficult did you find the X-target task?
3. How difficult did you find the 2-back task?
4. How important was it for you to perform well on the X-target task?
5. How important was it for you to perform well on the 2-back task?
6. How insecure, discouraged, irritated, stressed or annoyed were you during the X-target task?
7. How insecure, discouraged, irritated, stressed or annoyed were you during the 2-back task?
8. Did you find the X-target task distracted you from the pain?
9. Did you find the 2-back task distracted you from the pain?
10. Did you use any strategies or techniques to cope with the pain? If so, please describe:
11. Did you use any strategies or techniques for the 2-back task? If so, please describe:
12. Is there anything else you would like to tell us about your experiences or about the experiment?

**References**

Buhle, J., and Wager, T. D. (2010). Performance-dependent inhibition of pain by an executive working memory task: *Pain* 149, 19–26. doi:10.1016/j.pain.2009.10.027.

Lautenbacher, S., Peters, J. H., Heesen, M., Scheel, J., and Kunz, M. (2017). Age changes in pain perception: A systematic-review and meta-analysis of age effects on pain and tolerance thresholds. *Neuroscience & Biobehavioral Reviews* 75, 104–113. doi:10.1016/j.neubiorev.2017.01.039.

Legrain, V., Crombez, G., Verhoeven, K., and Mouraux, A. (2011). The role of working memory in the attentional control of pain. *PAIN®* 152, 453–459.

Rischer, K. M., González‐Roldán, A. M., Montoya, P., Gigl, S., Anton, F., and Meulen, M. (2020). Distraction from pain: The role of selective attention and pain catastrophizing. *Eur J Pain*, ejp.1634. doi:10.1002/ejp.1634.

Schmiedek, F., Li, S.-C., and Lindenberger, U. (2009). Interference and facilitation in spatial working memory: age-associated differences in lure effects in the n-back paradigm. *Psychology and aging* 24, 203.

Stanislaw, H., and Todorov, N. (1999). Calculation of signal detection theory measures. *Behavior research methods, instruments, & computers* 31, 137–149.

# 
