# Supplementary material for: Better Executive Functions Are Associated With More Efficient Cognitive Pain Modulation in Older Adults: An fMRI Study
Source: Front Aging Neurosci. 2022 Jul 7;14:828742. doi: 10.3389/fnagi.2022.828742 (PMC9302198; doi:10.3389/fnagi.2022.828742)
Supplement: Supplementary file 17 [file Data_Sheet_2.DOCX]

Supplementary Material

# Figure legends

**Figure S1.** The influence of negative pain-related cognitions on the neural distraction effect.

(A) Neural distraction effect across groups; (B) neural distraction effect for young adults > older adults. Note that figures are based on a t-test model (*p*(unc) < .005, *k* ≥ 10) and not a flexible factorial model and clusters may therefore slightly deviate from the clusters reported in the manuscript.

**Figure S2.** The influence of medication intake on the neural distraction effect.

(A) Neural distraction effect across groups; (B) neural distraction effect for young adults > older adults. Medication intake was coded as a binary variable (0: no regular intake of medication; 1: regular intake of medication). Figures are based on a t-test model (*p*(unc) < .005, *k* ≥ 10) and not a flexible factorial model and clusters may therefore slightly deviate from the clusters reported in the manuscript.

**Figure S3.** The influence of positive affect on the neural distraction effect.

(A) Neural distraction effect across groups; (B) neural distraction effect for young adults > older adults. Note that figures are based on a t-test model (*p*(unc) < .005, *k* ≥ 10) and not a flexible factorial model and clusters may therefore slightly deviate from the clusters reported in the manuscript.
