# Supplementary figures and images for: Better Executive Functions Are Associated With More Efficient Cognitive Pain Modulation in Older Adults: An fMRI Study
Source: Front Aging Neurosci. 2022 Jul 7;14:828742. doi: 10.3389/fnagi.2022.828742 (PMC9302198; doi:10.3389/fnagi.2022.828742)

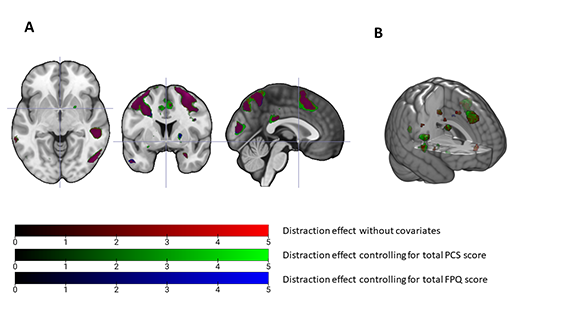

Supplement: Supplementary file 18 [file Image_1.TIF]

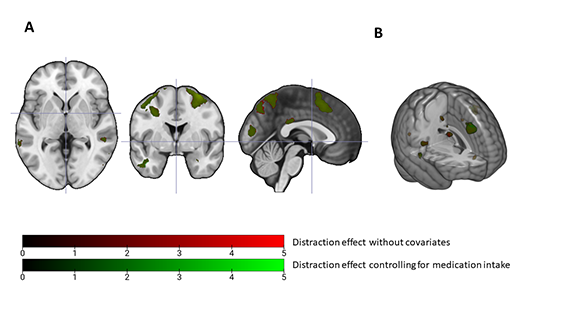

Supplement: Supplementary file 19 [file Image_2.TIF]

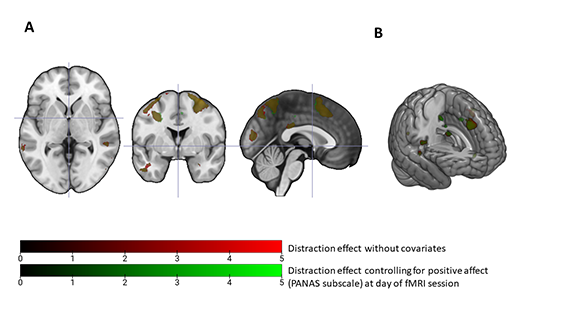

Supplement: Supplementary file 20 [file Image_3.TIF]
